# Predictions not commands: active inference in the motor system

**DOI:** 10.1007/s00429-012-0475-5

**Published:** 2012-11-06

**Authors:** Rick A. Adams, Stewart Shipp, Karl J. Friston

**Affiliations:** 1 The Wellcome Trust Centre for Neuroimaging, Institute of Neurology, University College London, 12 Queen Square, London, WC1N 3BG, UK; 2 UCL Institute of Ophthalmology, University College London, Bath Street, London, EC1V 9EL UK

**Keywords:** Active inference, Free energy, Hierarchy, Motor control, Reflexes

## Abstract

The descending projections from motor cortex share many features with top-down or backward connections in visual cortex; for example, corticospinal projections originate in infragranular layers, are highly divergent and (along with descending cortico-cortical projections) target cells expressing NMDA receptors. This is somewhat paradoxical because backward modulatory characteristics would not be expected of driving motor command signals. We resolve this apparent paradox using a functional characterisation of the motor system based on Helmholtz’s ideas about perception; namely, that perception is inference on the causes of visual sensations. We explain behaviour in terms of inference on the causes of proprioceptive sensations. This explanation appeals to active inference, in which higher cortical levels send descending proprioceptive *predictions*, rather than motor *commands*. This process mirrors perceptual inference in sensory cortex, where descending connections convey *predictions*, while ascending connections convey *prediction errors*. The anatomical substrate of this recurrent message passing is a hierarchical system consisting of functionally asymmetric driving (ascending) and modulatory (descending) connections: an arrangement that we show is almost exactly recapitulated in the motor system, in terms of its laminar, topographic and physiological characteristics. This perspective casts classical motor reflexes as minimising prediction errors and may provide a principled explanation for why motor cortex is agranular.

## Introduction

This paper tries to explain the functional anatomy of the motor system from a theoretical perspective. In particular, we address the apparently paradoxical observation that descending projections from the motor cortex are, anatomically and physiologically, more like backward connections in the visual cortex than the corresponding forward connections (Shipp 2005). Furthermore, there are some unique characteristics of motor cortex, such as its agranular cytoarchitecture, which remain unexplained. We propose that these features of motor projections are consistent with recent formulations of motor control in terms of *active inference*. In brief, we suggest that if sensory systems perform hierarchal perceptual inference, where descending signals are predictions of sensory inputs, then the functional anatomy of the motor system can be understood in exactly the same way, down to the level of classical motor reflex arcs. We develop this argument in five sections.

In the first section, we review the concept of perceptual inference from a Helmholtzian perspective, and describe how it can be instantiated by minimising prediction error using a hierarchical generative model. This treatment leads to the established notion of predictive coding in visual synthesis. Predictive coding schemes suggest that ascending and descending connections in cortical hierarchies must have distinct anatomical and physiological characteristics, which are remarkably consistent with empirical observations. In the second section, we introduce active inference as a generalisation of predictive coding, in which movement is considered to suppress proprioceptive prediction error. We discuss how active inference could have important implications for the organisation of the motor system, and illustrate the implicit mechanisms using the classical ‘knee-jerk’ reflex. The active inference view differs from the conventional (computational) views of motor control in conceptual and anatomical terms. Conceptually, under active inference, *predictions* about proprioceptive input are passed down the hierarchy; not motor *commands*. Anatomically, descending or efferent connections in active inference should be of the modulatory backward-type. Conversely, conventional motor control schemes would predict that descending motor command signals should be of the driving forward-type.

In the third section, we describe forward-type ascending and backward-type descending connections in the visual system, and use these features to furnish ‘tests’ for forward and backward connections in the motor system. In the subsequent section, we apply these tests to central and peripheral connections in the motor hierarchy, and find that descending connections are backward-type, and ascending connections are forward-type. This means the motor system conforms to the predictions of active inference. In the final section, we discuss the implications of this characterisation of the motor system, with a particular focus on the fact that primary motor cortex lacks a granular cell layer. Before we begin, we must clarify our nomenclature.

This paper refers to extrinsic connections between cortical areas (and subcortical structures and the spinal cord) as afferent, efferent, ascending, descending, forward, backward, driving and modulatory. We use ‘ascending’ (resp. afferent) and ‘descending’ (resp. efferent) in reference to the hierarchical *direction* of corticocortical and corticofugal projections: towards and away from high-level (association) cortex, respectively. We use ‘forward’ and ‘backward’ to describe the *characteristics* of projections, which can be laminar, topographic or physiological. For example, physiologically, ‘forward’ projections are ‘driving’ while ‘backward’ projections are ‘modulatory’. In sensory systems, ascending projections have forward-type, driving characteristics, and descending projections have backward-type, modulatory characteristics. This relationship does not necessarily hold in the motor system. The aim of this paper is to establish whether ‘descending’ motor connections are ‘forward’ or ‘backward’ and understand this designation in functional terms. If the theory behind active inference is broadly correct, then all projections of ‘ascending’ *direction* will have ‘forward’ *characteristics*, because their *function* is to convey prediction errors. Conversely, all projections of ‘descending’ *direction* will have ‘backward’ *characteristics*, because their *function* is to convey predictions.

We stress that we are not looking to impose an either/or classification upon every projection in the nervous system as regards ascending versus descending, forward versus backward and prediction error versus prediction. These are false partitions: for example—regarding the direction of projections—hierarchies also contain lateral connections (that are neither ascending nor descending, and with intermediate anatomical and physiological characteristics). Regarding the function of projections—not every projection in a predictive coding hierarchy conveys either a prediction or a prediction error: for example, the information carried by primary sensory afferents only becomes a prediction error signal once it encounters a prediction (which may be at the thalamus or in the spinal cord; see Fig. 9).

## Perception and predictive coding

Hermann von Helmholtz was the first to propose that the brain does not represent sensory images per se, but the *causes* of those images and, as these causes cannot be perceived directly, they must be inferred from sensory impressions (Helmholtz 1860/1962). In his study of optics, he noted that the richness of the brain’s visual perceptions contrasted with the signals coming from retinal nerves, which he felt could only differ in hue, intensity and retinal position. From these signals, the brain is able to perceive depth and spatial position, and maintain the size and colour constancy of objects. Helmholtz summarised this as, “We always think we see such objects before us as would have to be present in order to bring about the same retinal images”—we perceive the world as it is, and not as it is sensed. He concluded that to derive the causes of a retinal image from the image itself, the brain must perform unconscious inference.

How might such inferences be performed? What follows is a précis of arguments covered in depth elsewhere (Friston 2003). As Helmholtz pointed out, perception entails recognising the causes of sensation. In order to perceive, therefore, the brain must embody a *generative model* of how causes generate sensations. By simply inverting such a model (such that sensations generate causes), it can infer the most likely causes of its sensory data. The problem is that there are a multitude of interacting causes that give rise to the same sensory impressions. In vision, for instance, both object size and distance from the observer affect retinal image size. In these cases, straightforward inversion of the forward model becomes an ill-posed problem.

The solution to this ill-posed problem is to use a generative (forward) model that contains prior beliefs about how causes interact: e.g. that objects maintain a constant size irrespective of their distance from the observer. This inferential process is fundamentally Bayesian, as it involves the construction of a posterior probability density from a prior distribution over causes and sensory data. The brain cannot generate all of its prior beliefs de novo; instead it must estimate them from sensory data, which calls for *empirical* Bayes. Empirical Bayes uses a hierarchical generative model, in which estimates of causes at one level act as (empirical) priors for the level below. In this way, the brain can recapitulate the hierarchical causal structure of the environment: for example, the meaning of a phrase (encoded in semantic areas) predicts words (encoded in lexical areas), which predicts letters (encoded in ventral occipital areas), which predict oriented lines and edges (encoded in visual areas). All these hierarchically deployed explanations for visual input are internally consistent and distributed at multiple levels of description, where higher levels provide empirical priors that finesse the ill-posed inversion of the brain’s generative model.

A hierarchical generative model can be used to approximate the causes of sensory input by minimising the difference between the observed sensory data and the sensory data predicted or generated by the model (and indeed differences at all higher levels). These differences are known as *prediction error*, and the inversion scheme is generally called *predictive coding* (Rao and Ballard 1999). In predictive coding, backward projections from one hierarchical level to its subordinate level furnish predictions of the lower level’s representations, while reciprocal forward projections convey prediction errors that report the difference between the representation and the prediction (Mumford 1994). Error signals received by the higher level are then used to correct its representation so that its predictions improve. This recurrent exchange of signals continues until prediction error is minimised, at which point the hierarchical representation becomes a Bayes-optimal estimate of the (hierarchical) causes of sensory input.

The idea that the brain uses a predictive coding scheme has become increasingly popular, as evidence for such a scheme has accumulated in various modalities; e.g. Rao and Ballard (1999); Pessiglione et al. (2006); Henson and Gagnepain (2010); McNally et al. (2011); Rauss et al. (2011). In summary, predictive coding schemes suggest that descending predictions are subtracted from sensory input to generate an ascending prediction error, which corrects the prediction. This subtraction must be effected by local circuitry: the backward connections that carry descending predictions, like all long-range corticocortical (*extrinsic*) connections, originate in pyramidal cells and are excitatory. It is therefore generally assumed that the suppression of prediction error units is mediated by inhibitory interneurons (whose *intrinsic* connections are confined to each hierarchical level). The action of backward connections on layer 6 could be one such mechanism, as optogenetic manipulation of layer 6 pyramidal neurons in mouse V1 by Olsen et al. (2012) has demonstrated that excitation of layer 6 exerts a suppressive effect on neural activity in layers 2–5 (apart from fast-spiking inhibitory neurons in these layers, that showed enhanced activity). The sign-reversal effected by this backward pathway is clearly consistent with the tenets of predictive coding. Another potential mechanism for the suppression of prediction error is an inhibitory action of layer 1 activation on layer 2/3 pyramidal neurons (Shlosberg et al. 2006). Additional findings from non-invasive human studies suggest that top-down influences suppress overall activity in lower areas, when that activity can be predicted (Murray et al. 2002, 2006; Harrison et al. 2007; Summerfield et al. 2008, 2011). This suppression has been proposed as the basis of repetition suppression and phenomena such as the mismatch negativity in electrophysiology (Garrido et al. 2009; Vuust et al. 2009).

If the brain implements predictive coding, then its functional architecture ought to have particular attributes. These include: (1) a hierarchical organisation with (2) reciprocal connections between areas (conveying predictions and prediction errors) that are (3) divergent (because a cause has multiple consequences) and (4) functionally asymmetrical. The functional asymmetry is important because descending predictions have to embody nonlinearities in the generative model (e.g. to model visual occlusion) that require them to interact or modulate each other, whereas ascending connections that drive higher representations do not. These attributes are indeed characteristic of cortical architectures (Friston 2005). The functional asymmetry of ascending and descending connections is a critical issue for this paper, to which we shall return in the next section.

## Active inference, predictive coding and reflexes

So far we have discussed hierarchical models as they relate to perceptual inference, but we have made no reference to motor control. Before doing so, we must turn to a wider theory under which predictive coding can be subsumed: the free energy principle. This principle has been described extensively elsewhere (e.g. Friston et al. 2006; Friston 2010), and is summarised below. In brief, we will see that the Helmholtzian inference and predictive coding are only one side of the coin, in that action or behaviour also suppresses prediction errors. This rests on equipping the brain with motor reflexes that enable movement to suppress (proprioceptive) prediction errors. The free energy principle itself explains why it is necessary to minimise prediction errors.

Free energy is a concept borrowed from statistical physics. It is a quantity that bounds the *surprise* (negative log probability) of some (sensory) data, given a model of how those data were generated. The free energy principle explains how self-organising systems (like the brain) maintain their sensory states within physiological bounds, in the face of constant environmental flux. Such systems are obliged to minimise their sensory surprise, as this maximises the probability of remaining within physiological bounds (by definition). Although organisms cannot evaluate surprise directly, they can minimise a bound on surprise called (variational) free energy. Crucially, under some simplifying assumptions, free energy corresponds to prediction error. This is intuitive, in the sense that we are only surprised when our predictions are violated.

**Fig. 1:**
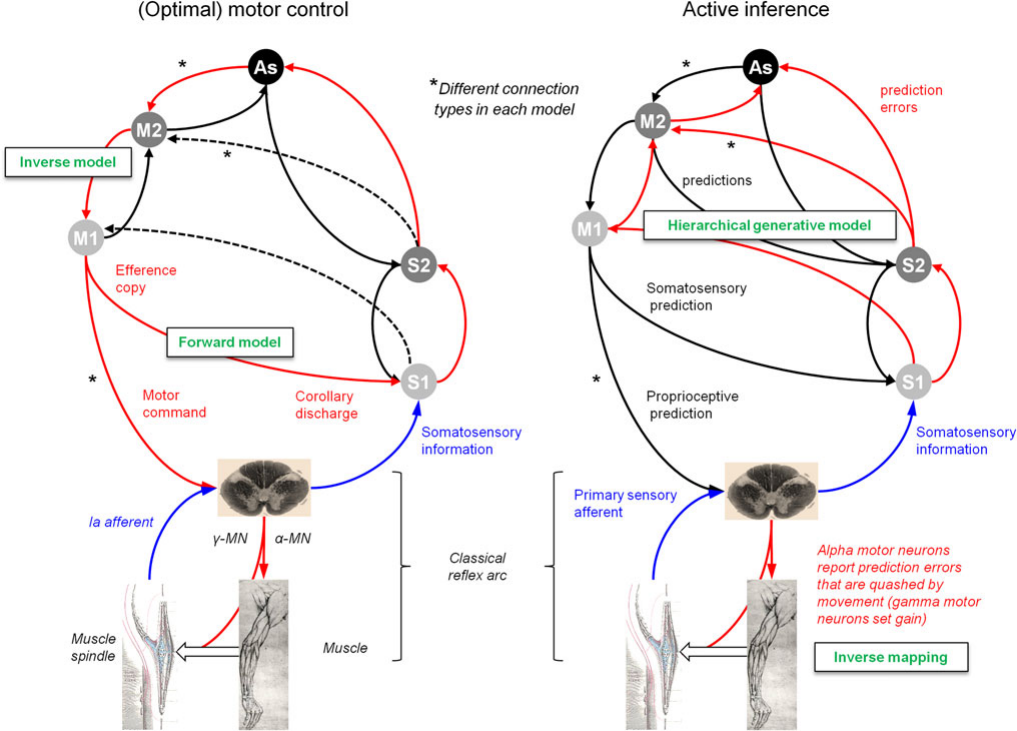
Motor control, optimal control and active inference: these simplified schematics ignore the contributions of spinal circuits and subcortical structures; and omit many hierarchical levels (especially on the sensory side). *M1*, *S1*, *M2* and *S2* signify primary and secondary motor and sensory cortex (S2 is area 5, not ‘SII’), while *As* signifies prefrontal association cortex. *Red arrows* denote driving ‘forward’ projections, and *black arrows* modulatory ‘backward’ projections. Afferent somatosensory projections are in *blue*. α-MN and γ-MN signify alpha- and gamma motor neuron output. The *dashed black arrows* in the optimal control scheme show what is different about optimal control compared with earlier serial models of the motor system: namely, the presence of sensory feedback connections to motor cortices. Under the active inference (predictive coding) scheme, all connections are reciprocal, with backward-type descending connections and forward-type ascending connections. They are descending from motor to sensory areas because the motor areas are above somatosensory areas in the hierarchy (see Fig. 4a). *Anatomical implications* The motor control and active inference models have identical connection types in the sensory system, but opposite connection types in the motor system (examples are indicated with *asterisks*). The nature of these connections should therefore disambiguate between the two models. The active inference model predicts descending motor connections should be backward-type, while conventional motor control schemes require the descending connections to convey driving motor commands. *Predictions and prediction errors* In the active inference scheme, backward connections convey predictions, and the forward connections deliver prediction errors. In the motor control scheme, the descending forward connections from M1 convey motor commands computed by an inverse model for generating movements and efference copy required by a forward model, for predicting its sensory consequences. *The classical reflex arc* The active inference model illustrates how the classical reflex arc performs an inverse mapping from sensory predictions to action (motor commands). The (classical) reflex arcs we have in mind are a nuanced version of Granit’s (1963) proposal that, in voluntary movements, a reference value is set by descending signals, which act on both the alpha and gamma motor neurons—known as *alpha*-*gamma coactivation* (Matthews 1959; Feldman and Orlovsky 1972). In this setting, the rate of firing of alpha motor neurons is set (by proprioceptive prediction errors) to produce the desired (predicted) shortening of the muscle, though innervation of extrafusal muscle fibres; while the rate of firing of gamma motor neurons optimises the sensitivity or gain of muscle spindles, though innervation of intrafusal muscle fibres. Note the emphasis here is on alpha motor neurons as carrying proprioceptive prediction errors derived from the comparison of descending predictions (about movement trajectories) and primary afferents (see Fig. 2). In this setting, gamma motor neurons are considered to provide context-sensitive modulation or gain of primary afferents (e.g. ensure they report changes in muscle length and velocity within their dynamic range). *Forward and inverse models* Conventional (computational) motor control theory uses the notion of forward–inverse models to explain how the brain generates actions from desired sensory states (the *inverse model*) and predicts the sensory consequences of action (the *forward model*). In these schemes, the inverse model has to generate a motor command from sensory cues—a complex transformation—and then a forward model uses an *efference copy* of this command to generate a predicted proprioceptive outcome called *corollary discharge* (Wolpert and Kawato 1998). In active inference a forward or *generative model* generates both proprioceptive and sensory predictions—a simple transformation—and an inverse mapping converts a proprioceptive prediction into movement. This is a relatively well-posed problem and could be implemented by spinal reflex arcs (Friston et al. 2010). In the terminology of this paper, optimal control’s inverse *model* maps from an extrinsic frame to an intrinsic frame and from an intrinsic frame to motor commands. The inverse *mapping* in active inference is simply from the intrinsic frame to motor commands. This figure omits the significant contribution of the cerebellum to the forward model

**Fig. 2:**
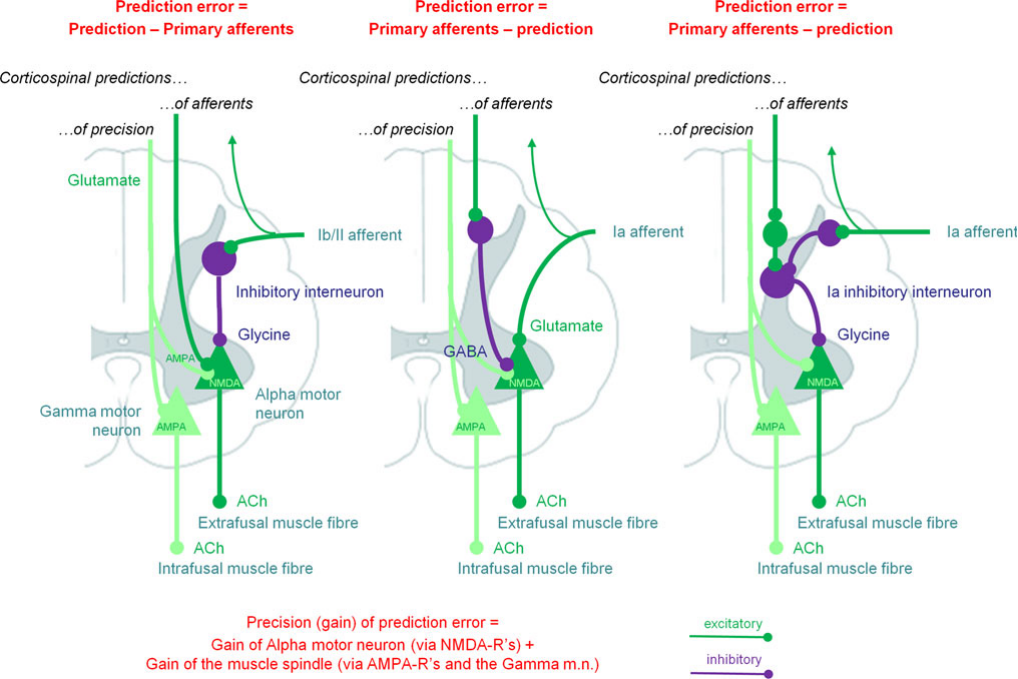
Generation of spinal prediction errors and the classical reflex arc. This schematic provides examples of spinal cord circuitry that are consistent with its empirical features and could mediate proprioceptive predictions. They all distinguish between descending proprioceptive predictions of (Ia and Ib) primary afferents and predictions of the precision of the ensuing prediction error. Predictions of precision optimise the gain of prediction error by facilitating descending predictions (through NMDA receptor activation) and the afferents predicted (through gamma motor neuron drive to intrafusal muscle fibres). This necessarily entails alpha-gamma coactivation and renders descending predictions (of precision) facilitatory. The prediction errors per se are simply the difference between predictions and afferent input. The *left panel* considers this to be mediated by convergent monosynaptic (AMPA-R mediated) descending projections (‘CM’ neurons) and inhibition, mediated by the inhibitory interneurons of Ib (Rudomin and Schmidt 1999) or II (Bannatyne et al. 2006) afferents. The *middle and right panels* consider the actions of Ia afferents, which drive (or disinhibit) alpha motor neurons, in opposition to (inhibitory) descending predictions. The *middle panel* is based on Hultborn et al. (1987) and the *right panel* on Lindström (1973). Note that corticospinal neurons synapse directly with spinal motor neurons and indirectly via interneurons (Lemon 2008). When a reflex is elicited by stretching a tendon, sudden lengthening of the (fusimotor) muscle spindle stretch receptors sends proprioceptive signals (via primary sensory Ia neurons) to the dorsal root of the spinal cord. These sensory signals excite (disinhibit) alpha motor neurons, which contract (extrafusal) muscle fibres and return the stretch receptors to their original state. The activation of alpha motor neurons by sensory afferents can be *monosynaptic* or *polysynaptic*. In the case of monosynaptic (simple) reflex arcs (*middle panel*), a prediction error is generated by inhibition of the alpha motor neurons by descending predictions from upper motor neurons. In polysynaptic (spinal) reflexes, Ia inhibitory interneurons may report prediction errors (*right panel*). Ia inhibitory interneurons are inhibited by sensory afferents (via glycine) and this inhibition is countered by descending corticospinal efferents (Lindström 1973). In this polysynaptic case, reflex muscle fibre contractions are elicited by disinhibition of alpha motor neuron drive. Crucially, precisely the same muscle contractions can result from changes in descending (corticospinal) predictions. This could involve suspension of descending (glutamatergic) activation either of presynaptic inhibition of Ia afferents (Hultborn et al. 1987; reviewed by Rudomin and Schmidt 1999)—not shown—or of Ia inhibitory interneurons, and disinhibition of alpha motor neuron activity. The ensuing mismatch or prediction error is resolved by muscle contraction and a reduction in stretch receptor discharge rates. In both reflexes and voluntary movement, under active inference the motor system is enslaved to fulfil descending proprioceptive predictions. As Feldman (2009) notes, “posture-stabilizing mechanisms (i.e. classical reflex arcs) do not *resist* but *assist* the movement” [italics in original]: threshold control theory does this by changing the threshold position, active inference by changing proprioceptive predictions. The key aspect of this circuitry is that it places descending corticospinal efferents and primary afferents in opposition, through inhibitory interneurons. The role of inhibitory interneurons is often portrayed in terms of a reciprocal inhibitory control of agonist and antagonist muscles. However, in the setting of predictive coding, they play a simpler and more fundamental role in the formation of prediction errors. This role is remarkably consistent with computational architectures in the cortex and thalamus: for example, top-down projections in the sensory hierarchies activate inhibitory neurons in layer 1 which then suppress (superficial) pyramidal cells, thought to encode prediction error (Shlosberg et al. 2006). Note that there are many issues we have ignored in these schematics, such as the role of polysynaptic transformations, nonlinear dendritic integration, presynaptic inhibition by cutaneous afferents, neuromodulatory effects, the role of Renshaw cells, and other types of primary afferents
- If both systems are minimising prediction error, descending hierarchical projections in the motor cortex should share the laminar, topographic and physiological characteristics of backward connections in exteroceptive (sensory) systems.

The brain can minimise prediction error in one of two ways. It can either change its predictions to better cohere with sensory input, or change the sampling of the environment such that sensory samples conform to predictions. The former process corresponds to perceptual inference—discussed in the previous section as predictive coding—the latter to *action*: together, they constitute ‘active inference’ (Friston et al. 2010). The free energy principle thus dictates that the perceptual and motor systems should not be regarded as separate but instead as a single active inference machine that tries to predict its sensory input in all domains: visual, auditory, somatosensory, interoceptive and, in the case of the motor system, *proprioceptive* (cf. Censor et al.’s (2012) analysis of common learning mechanisms in the sensory and motor systems). In what follows, we look at the implications of this for the somatomotor system, in which we include sensory afferents relevant to motor control (e.g. proprioceptors), all motor efferents, and associated cortical and subcortical systems. Active inference has the following important implications for the somatomotor system (also see Fig. 1):

- In common with the rest of the central nervous system, it should embody a hierarchical generative model that enables the minimisation of prediction errors by its (descending) predictions.
- Descending messages in the somatomotor system are therefore *predictions of proprioceptive input* and not motor commands.
- In the somatosensory system, predictions of sensory input are corrected by prediction errors in the usual way during exteroception (although note that some of these somatosensory predictions will come from the somatomotor system, e.g. cutaneous sensations during gripping—see the “Discussion”). In the somatomotor system, however, proprioceptive predictions should not be *corrected* but *fulfilled*, by the automatic peripheral transformation of proprioceptive prediction errors into movement. The neuronal encoding of predictions—in terms of the activity of specific neuronal populations—and the transformations—mediated through synaptic connections—conform to the neurobiologically plausible schemes considered for predictive coding in the brain (for details, see Friston et al. 2010). A proprioceptive prediction error can be generated at the level of the spinal cord by the comparison of proprioceptive predictions (from motor cortex) and proprioceptive input. Sources of proprioceptive input include muscle spindles (via Ia and II afferents), Golgi tendon organs (via Ib afferents), and articular and cutaneous receptors. The prediction error can then activate the motor neuron to contract the muscle in which the spindles—or other receptors—are sited: this is the classical reflex arc (Figs. 1, 2). In short, peripheral proprioceptive prediction errors are (or become) motor commands.

The second point above raises the question: what exactly is the difference between a top-down prediction of proprioceptive input and a top-down motor command? In principle a motor command is a signal that drives a muscle (motor unit) and should not show context specificity: the command to one motor unit should not depend upon the commands to another. In contrast, a prediction of proprioceptive input encodes the *consequences* of a movement rather than its *cause.* 1 Given that these consequences are a nonlinear function of their causes, the proprioceptive predictions for several motor units should be interdependent. For example, proprioceptive consequences are modulated by the current position of the limb. M1 efferents do in fact have the characteristics of proprioceptive predictions: stimulation of points in M1 activates either biceps or triceps differentially, according to the degree of flexion of the monkey’s arm (Graziano 2006). Furthermore, prolonged (500 ms) stimulation of M1 causes movement of a monkey’s arm to specific locations, no matter what position the arm started in (Graziano 2006). This stimulation regime is controversial (Strick 2002), as it is non-physiological and stimulus-driven activity has been shown to ‘hijack’ all activity in the resulting M1 output (Griffin et al. 2011). Nevertheless, one can still argue that under this non-physiological stimulation, the M1 layer 5 pyramidal cells’ output encodes the goal of the movement and not the motor commands for generating that movement (because the necessary commands to reach a given location would be different at different starting positions). Whether *physiological* M1 activity can be said to encode goals or motor commands is reviewed in the “Discussion”.

In brief, under active inference, descending signals do not enact motor commands directly, but specify the desired consequences of a movement.^2^ These descending signals are either predictions of proprioceptive input or predictions of precision or gain (see Fig. 2 and the “Discussion” for explication of the latter).

Our focus in this paper is on the functional anatomy of the motor system, considered in light of active inference. Although we have stressed the importance of hierarchical message passing in predictive coding, we shall not consider in detail where top-down predictions and (empirical) priors come from. Priors in the motor system are considered to be established in the same way as in perceptual systems: some would be genetically specified and present from birth (e.g. innate reflexes), while most would be learned during development. The easiest way to demonstrate their existence empirically is to show their effects on evoked responses to stimuli; i.e. their contribution to prediction error responses. In perception, it has been shown that the mismatch negativity response is best characterised as that of a predictive coding network to a change in a stimulus about which prior beliefs have been formed (Garrido et al. 2009). There are myriad of other examples of how learning priors about stimuli changes the responses they evoke: e.g. for visual (Summerfield et al. 2008; Summerfield and Koechlin 2008), auditory (Pincze et al. 2002), and somatosensory (Akatsuka et al. 2007) stimuli. As the brain learns these changing probabilities, they can be expressed in the motor domain as increased speed and accuracy of motor responses (den Ouden et al. 2010). There is also a literature which demonstrates the effects of learning priors on single cell responses in electrophysiology (e.g. Rao and Ballard 1999; Spratling 2010).

The idea that the motor cortex specifies consequences of, rather than instructions for, movements is not a new one. More than half a century ago, Merton (1953) proposed the servo hypothesis, which held that descending motor signals activated gamma motor neurons, specifying the desired length of the muscle. This changed the sensitivity of their muscle spindles, thereby activating alpha motor neurons via the tonic stretch reflex, which causes the muscle to contract until its length reached the point specified by the gamma motor neurons. The servo hypothesis assumed that while the descending command remains constant, muscle length will also remain constant, because changes in load will be compensated for by the tonic stretch reflex. The servo hypothesis did not survive because gamma and alpha motor neurons were shown to activate simultaneously, not sequentially (Granit 1955), and the gain of the tonic stretch reflex was shown to be insufficient for maximal increases in muscle force with minimal displacement (Vallbo 1970).

The successor to the servo hypothesis is the equilibrium point hypothesis—or more properly, threshold control theory (Feldman and Levin 2009), which proposes that descending signals to both alpha and gamma motor neurons specify the relationship between muscle force and muscle length—by setting the threshold of the tonic stretch reflex—such that a given load will result in the muscle reaching the specific length at which its force matches the external load: the ‘equilibrium point’. For a constant descending signal, changes in this external load would result in predictable changes in muscle length, as it is the relationship between force and length which descending signals dictate, not the absolute length (unlike the servo hypothesis).

Threshold control theory and active inference are closely related and consensual in several respects. First, both eschew the complex calculation of motor commands by the central nervous system (CNS); instead, they merely ask the CNS to specify the sensory conditions under which motor commands should emerge—through the operation of classical reflex arcs. In threshold control theory, the sensory conditions specified by the CNS are the threshold positions at which muscles begin to be recruited in order to achieve a narrow subset of equilibrium points. In active inference, they are the sensory consequences of movement, which then undergo automatic peripheral transformation into motor commands.

Second, neither theory holds that redundancy problems in motor control require an optimality criterion to choose between competing trajectories (see Friston 2011 for further discussion). Third, both theories propose that the sensory conditions under which motor commands emerge are specified in an *extrinsic* frame of reference—as opposed to an intrinsic (muscle based) frame of reference. This enables top-down predictions about the consequences of movement in other sensory modalities, which can be regarded as corollary discharge. Crucially, this obviates the need for a complex (ill posed) transformation of efference copy from intrinsic to extrinsic frames (Feldman 2008).

There are two essential differences between the theories. First, active inference is grounded in predictive coding, and therefore holds that descending signals are predictions of the sensory consequences of movement. This is in contrast to threshold control theory, which does not predict proprioceptive or torque-related states—the threshold position is not the movement ‘prediction’ and deviation from this position is not a ‘prediction error’—instead, the threshold position is a tool for the production of actions and the interpretation of (otherwise ambiguous) kinaesthetic information.

Second, in threshold control theory, changing descending signals lead (via changing threshold positions) to new equilibrium points that are defined in terms of position and torque. In active inference, descending signals specify sensory trajectories whose fixed point is the equilibrium point; i.e. the dynamics of the movement (including velocity, acceleration, jerk, etc), not just the position and torque at an end point (Friston et al. 2010).

The last of the four implications of active inference for the nervous system listed above motivates the following hypothesis, which we address in the remainder of this paper.

Under active inference, descending projections in the motor hierarchy convey proprioceptive predictions and therefore should have comparable laminar, topographic and physiological characteristics as backward projections in exteroceptive (e.g. visual) hierarchies.

Conversely, conventional models of the somatomotor system, as exemplified in the motor control literature (Shadmehr et al. 2010), consider descending connections to deliver driving command signals and therefore to be of the forward type. The conventional motor control model is taken here to treat the brain as an input–output system that mediates stimulus–response mappings—in which sensory signals are passed forwards to sensory to association to motor cortex and then to the spinal cord and cranial nerve nuclei as motor commands. In computational motor control this usually involves the use of *forward* and *inverse* models, where the inverse model supplies the motor command and the forward model converts efference copy into sensory predictions (Wolpert and Kawato 1998). These predictions are used to optimise the estimated state of the motor plant required by the inverse model (see Fig. 1 for a schematic that compares active inference and motor control schemes).

In the last 10 years, optimal motor control has become a dominant model of motor control (Scott 2004). This model was based on influential work by Todorov and Jordan (2002, 2004), who showed the selective use of sensory feedback to correct deviations that interfere with task goals could account for several unexplained effects in motor control, such as the variability of task-irrelevant movement qualities. The idea that motor cortex could use sensory feedback contrasted with the earlier purely ‘feed-forward’ serial model of motor control (see Fig. 1). The optimal control model has some commonalities with the active inference view, in that both propose that sensory inputs to motor cortex finesse its output: in optimal control theory, these inputs are state estimates that the optimal controller uses to optimise motor commands. Under active inference, these inputs are proprioceptive and somatosensory prediction errors, which a forward model uses to derive proprioceptive predictions. However, there are profound differences between the two: a crucial theoretical difference—explained at length in Friston (2011)—is that optimal control models generate optimal motor commands by minimising a cost function associated with movement. In active inference schemes, the cost functions are replaced by prior beliefs about desired trajectories in extrinsic frames of reference, which emerge naturally during hierarchical perceptual inference.

Of interest in the present context, is an important difference between the signals descending the spinal cord in the two models: under active inference these are proprioceptive predictions, whereas in optimal control—as in earlier serial models—these signals are motor commands. In neurobiological terms, predictions must have modulatory or non-linear context-dependent (backward-type) properties, whereas commands must have driving, linear, context-independent (forward-type) properties. We assume here, that predictions (or commands) are communicated through the firing rate modulation of descending efferents of upper motor neurons in M1. The key difference between predictions and commands is that the former have yet to be converted (inverted) into command signals that fulfil the predictions (goals). This conversion necessarily entails context-sensitivity—for example, producing different command signals at the spinal level, depending upon limb position. Another difference lies in the nature of the sensory input to motor cortex: under active inference, these ascending signals must be sensory *prediction errors* (in predictive coding architectures, ascending signals cannot be predictions), whereas in optimal control these inputs to the optimal controller (inverse model) are state estimates, i.e. *sensory predictions*.

The analysis above means that characterising somatomotor connections as forward or backward should disambiguate between schemes based on active inference and optimal motor control. In the next section, we describe the characteristics of forward (ascending) and backward (descending) projections in sensory hierarchies, and then apply these characteristics as tests to motor projections in the subsequent section.

## Forward and backward connections

In this section, we review the characteristics of ascending and descending projections in the visual system, as this is the paradigmatic sensory hierarchy. The characteristics of ascending visual projections will become tests of forward projections (i.e. those conveying prediction errors) and the characteristics of descending visual projections will constitute tests of backward projections (i.e. those conveying predictions). These characteristics can be grouped into four areas; laminar, topographic, physiological and pharmacological (also see Table 1).

**Table 1:** Columns 2 and 4 summarise the characteristics of *forward* (driving) and *backward* (modulatory) connections in sensory cortex

Test	Forward connections in sensory cortex	Ascending connections in motor cortex	Backward connections in sensory cortex	Descending connections in motor cortex (and periphery)
Origin	Supra ≫ infragranular	Bilaminar (Supra > infragranular)	Infra > supragranular	Bilaminar (Supra > infragranular), but of a lower S:I ratio than the ascending connections*
Termination	Layer 4 (granular)	Multilaminar in higher motor areas; layer 3 in S1 to M1	Concentrated in layers 1 and 6, avoiding layer 4	Multilaminar, concentrated in layer 1 and avoiding lower layer 3 (or layer 4 in sensory cortex)
Axonal properties	Rarely bifurcate, patchy terminations	Not described	Commonly bifurcate, widely distributed terminations	Motor neurons innervate hundreds of muscle fibres in a uniform distribution; corticospinal axons innervate many motor neurons in different muscle groups
Vergence	Somatotopic, more segregated	S1 to M1 and peripheral proprioceptive connections to M1 are more somatotopic and segregated	Less somatotopic, more diffuse	M1 to periphery very divergent and convergent; cingulate, SMA and PMC to M1 less somatotopic
Proportion	Fewer	See descending column	Greater	Greater from M1 to the periphery, areas 6–4, F6 to F3, and CMAr to SMA/PMdr
Physiological and pharmacological properties	More driving in character (via non-NMDA-Rs)	S1 connections to M1 more driving than PMC connections; M1’s ascending input is via non-NMDA-Rs	More modulatory in character (projecting to supragranular NMDA-Rs)	NMDA receptors in supragranular distribution; 50 % of M1’s descending input is via NMDA-Rs; F5 has a powerful facilitatory effect on M1 outputs but is not itself driving

These are used as tests of the connection type of *ascending* (afferent) and *descending* (efferent) projections in motor cortex and the periphery. As can be seen from columns 3 and 5, ascending connections in motor cortex are forward (driving), and descending connections are backward (modulatory); the one exception (marked *) has some mitigating properties, as discussed in the text (see “Laminar characteristics” in “Motor projections”). This pattern is predicted by our active inference model of somatomotor organisation

### Laminar characteristics

The cerebral neocortex consists of six layers of neurons, defined by differences in neuronal composition (pyramidal or stellate excitatory neurons, and numerous inhibitory classes) and packing density (Shipp 2007). Layer 4 is known as the ‘internal granular layer’ or just ‘granular layer’ (due to its appearance), and the layers above and below it are known as ‘supragranular’ and ‘infragranular’, respectively. Since the late 1970s (e.g. Rockland and Pandya 1979), it has been known that extrinsic corticocortical (ignoring thalamocortical) connections between areas in the visual system have distinct laminar characteristics, which depend on whether they are ascending (forward) or descending (backward).

Felleman and Van Essen (1991) surveyed 156 corticocortical pathways and specified criteria by which projections could be classified as forward, backward or lateral. They defined forward projections as originating predominantly (i.e. >70 % cells of origin) in supragranular layers, or occasionally with a bilaminar pattern (meaning <70 % either supra- or infragranular, but excluding layer 4 itself). Forward projections terminate preferentially in layer 4. Backward projections are predominantly infragranular or bilaminar in origin with terminations in layers 1 and 6 (especially the former), and always evading layer 4 (see Table 1). Further refinements to this scheme suggest the operation of a ‘distance rule’, whereby forward and backward laminar characteristics become more accentuated for connections traversing two or more tiers in the hierarchy (Barone et al. 2000).

### Topographic characteristics

Salin and Bullier (1995) reviewed a large body of evidence concerning the microscopic and macroscopic topography of corticocortical connections, and how these structural properties contribute to their function; e.g. their receptive fields. In cat area 17, for example, <3 % of forward projecting neurons have axons which bifurcate to separate cortical destinations. Conversely, backward projections to areas 17 and 18 include as much as 30 % bifurcating axons (Bullier et al. 1984; Ferrer et al. 1992). A similar relationship exists in visual areas in the monkey (Salin and Bullier 1995).

**Fig. 3:**
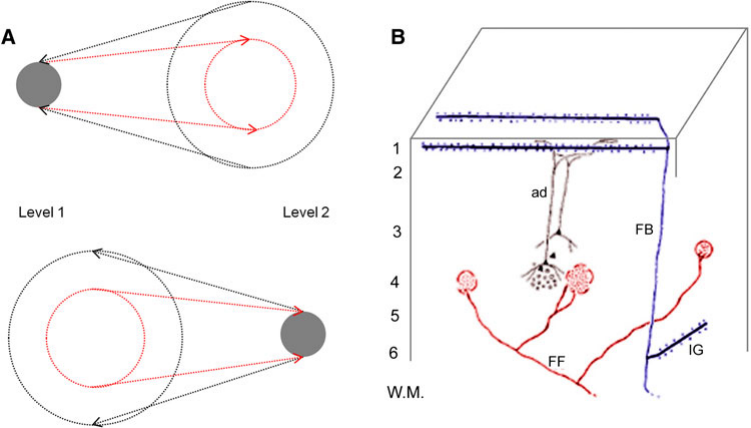
Topographic characteristics of forward and backward projections. **a** This schematic illustrates projections to and from a lower and higher level in the visual hierarchy (adapted from Zeki and Shipp 1988). *Red arrows* signify forward connections and *black arrows* backward connections. Note that there is a much greater convergence (from the point of view of neurons receiving projections) and divergence (from the point of view of neurons sending projections) in backward relative to forward connections. **b** This schematic is adapted from Rockland and Drash (1996), and illustrates the terminal fields of ‘typical’ forward (axon *FF* *red*) and backward (axon *FB* *purple*) connections in the visual system. *IG* represents infragranular collaterals of a backward connection, and *ad* an apical dendrite; cortical layers are *labelled on the left*. Note the few delimited arbours of terminals on the forward connection, and the widely distributed “wand-like array” of backward connection terminals

Rockland and Drash (1996) contrasted a subset of backward connections from late visual areas (TE and TF) to primary visual cortex with typical forward connections in the macaque. The forward connections concentrated their synaptic terminals in 1–3 arbours of around 0.25 mm in diameter, whilst backward connections were distributed over a “wand-like array” of neurons, with numerous terminal fields stretching over 4–10 mm, and in one case, 21 mm (Fig. 3b). This very diffuse pattern was only found in around 10 % of backward projections, but it was not found in any forward projections.

These microscopic properties of backward connections reflect their greater macroscopic divergence. Zeki and Shipp (1988) reviewed forward and backward connections between areas V1, V2 and V5 in macaques, and concluded that backward connections showed much greater convergence and divergence than their forward counterparts (Fig. 3a). This means that cells in higher visual areas project back to a wider area than that which projects to them, and cells in lower visual areas receive projections from a wider area than they project to. Whereas forward connections are typically patchy in nature, backward connections are more diffuse and, even when patchy, their terminals can be spread over a larger area than the deployment of neurons projecting to them (Shipp and Zeki 1989a, b; Salin and Bullier 1995). These attributes mean that visuotopy preserved in the forward direction is eroded in the backward direction, allowing backward projections to contribute significantly to the extra classical receptive field of a cell (Angelucci and Bullier 2003).

Salin and Bullier (1995) also noted that in the macaque ventral occipitotemporal pathway (devoted to object recognition), backward connections outnumber forward connections. Forward projections from the lateral geniculate nucleus (LGN) to V1 are outnumbered 20 to 1 by those returning in the opposite direction; and backward projections outweigh forward projections linking central V1 to V4, TEO to TE, and TEO and TE to parahippocampal and hippocampal areas. It is perhaps significant that backward connections should be so prevalent in the object recognition pathway, given the clear evolutionary importance of recognising objects and the fact that occluded objects are a classic example of nonlinearity, whose recognition may depend on top-down predictions (Mumford 1994).

### Physiological characteristics

Forward and backward connections in sensory systems have always been associated with ‘driving’ and ‘modulatory’ characteristics, respectively, though the latter physiological duality has lacked the empirical clarity of its anatomical counterpart, particularly for cortical interactions.

The simple but fundamental observation that visual receptive field size increases at successive tiers of the cortical hierarchy implies that a spatially restricted subset of the total forward input to a neuron is capable of driving it; evidently the same is not true, in general, of the backward connections. Experiments manipulating feedback (e.g. by cooling) found no effect upon spontaneous activity, and were generally consistent with the formulation that backward input might alter the way in which a neuron would respond to its forward, driving input, but did not influence activity in the absence of that driving input, nor fundamentally alter the specificity of the response (Bullier et al. 2001; Martinez-Conde et al. 1999; Przybyszewski et al. 2000; Sandell and Schiller 1982). Thus driving and modulatory effects could be defined in a somewhat circular, but logically coherent fashion.

The generic concept of driving versus modulatory also applies to the primary thalamic relay nuclei, where driving by forward connections implies an obligatory correlation of pre and post-synaptic activity (e.g. as measured by a cross-correlogram), that is barely detectable in backward connections (Sherman and Guillery 1998). LGN neurons, for instance, essentially inherit their receptive field characteristics from a minority of retinal afferents, whilst displaying a variety of subtler influences of cortical origin; these derive from layer 6 of V1, and modulate the level and synchrony of activity amongst LGN neurons. In vitro—in slice preparations—driving connections produce large excitatory postsynaptic potentials (EPSPs) to the first action potential of a series that diminish in size with subsequent action potentials (Li et al. 2003; Turner and Salt 1998). The effect is sufficiently discernible with just two impulses, and termed ‘paired-pulse depression’. It is also ‘all or none’—the magnitude of electrical stimulation can determine the probability of eliciting an EPSP, but not its size. Modulating connections, by contrast, have smaller initial EPSPs that grow larger with subsequent stimuli (i.e. ‘paired pulse facilitation’), and show a non-linear response to variations in stimulus magnitude. Both types of EPSP are blocked by antagonists of ionotropic glutamate receptors.

Much as the study of laminar patterns of termination imposed greater rigour on the concept of hierarchy, the in vitro properties offer a robust, empirical definition of driving and modulatory synaptic contacts (Reichova and Sherman 2004). The latter also use metabotropic glutamate receptors (mGluRs), which are not found in driving connections. More recent work has extended the classification from thalamic synapses to thalamocortical and corticocortical connections between primary and secondary sensory areas (Covic and Sherman 2011; De Pasquale and Sherman 2011; Lee and Sherman 2008; Viaene et al. 2011a, b, c). A crucial question for this work is the extent to which its in vitro findings are applicable in vivo, as several of its initial results are at odds with previous generalisations: not least the finding that forward and backward connections can have equal and symmetrical driving and modulatory characteristics, albeit between cortical areas that are close to each other in the cortical hierarchy. With respect to this question, there are at least three sets of considerations that deserve attention:

1. In vivo and in vitro physiologies are not identical (Borst 2010). Importantly, the paired-pulse investigations routinely add GABA blocking agents to the incubation medium, to avoid masking of glutamate excitation. In vitro conditions are further characterised, in general, by a higher concentration of calcium ions, and lower levels of tonic network activity.
2. The paired pulse effects are largely presynaptic in origin, and reflect the variability of transmitter release probability rather than the operational characteristics of the synapse in vivo (Beck et al. 2005; Branco and Staras 2009; Dobrunz and Stevens 1997). Due to the factors mentioned in (1), release probability is higher in vitro (Borst 2010).
3. The physiology of forward/backward connections will depend upon many factors—laminar distribution, the cell-types contacted, location of synapses within the dendritic arborisation, and the nature of postsynaptic receptors—in addition to the presynaptic release mechanisms.

Each one of these factors might constrain the ability of ‘drivers’ to drive in vivo. For instance, even in an in vitro system, tonic activity has been shown to switch corticothalamic driver synapses to a ‘coincidence mode’, requiring co-stimulation of two terminals to achieve postsynaptic spiking (Groh et al. 2008). We will therefore assume a distinction between driving and modulation in operational terms; i.e. as might be found in vivo (e.g. neuroimaging studies—see Büchel and Friston 1997). In the realm of whole-brain signal analysis, a related distinction can be drawn between linear (driving) and nonlinear (modulatory) frequency coupling (Chen et al. 2009).

We now consider the factors listed in (3) above and evidence linking nonlinear (modulatory) effects to backward connections, much of which depends on a closer consideration of the roles played by the different types of postsynaptic glutamate receptors:

### Pharmacological characteristics

Glutamate is the principal excitatory neurotransmitter in the cortex and activates both ionotropic and metabotropic receptors. Metabotropic receptor binding triggers effects with the longest time course, and is clearly modulatory in action (Pin and Duvoisin 1995). Spiking transmission is mediated by ionotropic glutamate receptors, classified according to their AMPA, kainate and NMDA agonists (Traynelis et al. 2010). These are typically co-localised, and co-activated, but profoundly different biophysically. AMPA activation is fast and stereotyped, with onset times <1 ms, and deactivation within 3 ms; recombinant kainate receptors have AMPA receptor-like kinetics, although they can be slower in vivo. NMDA currents, by contrast, are smaller but more prolonged: the onset and deactivation are one and two orders of magnitude slower, respectively.

Unlike non-NMDA receptors, NMDA receptors are both ligand-gated and voltage-dependent—to open their channel they require both glutamate binding and membrane depolarisation to displace the blocking Mg^2+^ ion. The voltage dependence makes NMDA transmission non-linear and the receptors function, in effect, as postsynaptic coincidence detectors. These properties may be particularly important in governing the temporal patterning of network activity (Durstewitz 2009). Once activated, NMDA receptors play a critical role in changing long-term synaptic plasticity (via Ca^2+^ influx) and increase the short-term gain of AMPA/kainate receptors (Larkum et al. 2004). In summary, NMDA receptors are nonlinear and modulatory in character, whereas non-NMDA receptors have more phasic, driving properties.

NMDA receptors (NMDA-Rs) are ubiquitous in distribution, and clearly participate in forward, intrinsic and backward signal processing. They occur, for instance, at both sensory and cortical synapses with thalamic relay cells (Salt 2002). The ratio of NMDA-R:non-NMDA-R synaptic current is not necessarily equivalent, however, and it is known to be greater at the synapses of backward connections in at least one system, the rodent somatosensory relay (Hsu et al. 2010). In the cortex, NMDA-R density can vary across layers, in parallel with certain other modulatory receptors (e.g. cholinergic, serotoninergic; Eickhoff et al. 2007). The key variable of interest may rather be the subunit composition of NMDA-Rs (NR1 and NR2). The NR2 subunit has four variants (NR2A–D), which possess variable affinity for Mg^2+^ and affect the speed of release from Mg^2+^ block, the channel conductance and its deactivation time. Of these the NR2B subunit has the slowest kinetics for release of Mg^2+^, making NMDA-R that contain the NR2B subunit the most nonlinear, and the most effective summators of EPSPs (Cull-Candy and Leszkiewicz 2004). In macaque sensory cortex, the NR2B subunit is densest in layer 2, followed by layer 6 (Muñoz et al. 1999)—the two cellular layers in which feedback terminates most densely (equivalent data for other areas is not available). Predictive coding requires descending non-linear predictions to negate ascending prediction errors, and interestingly, it seems that the inhibitory effects of backward projections to macaque V1 are mediated by NR2B-containing NMDA-R’s (Self et al. 2012). By contrast, layer 4 of area 3B, in particular, features a highly discrete expression of the NR2C subunit (Muñoz et al. 1999), which has faster Mg^2+^ kinetics (Clarke and Johnson 2006); in rodent S1 (barrel field) intrinsic connections between stellate cells in layer 4 have also been demonstrated to utilise NMDA-R currents that are minimally susceptible to Mg^2+^ block, and these cells again show high expression of the NR2C subunit (Binshtok et al. 2006). In general, therefore, the degree of nonlinearity conferred on the NMDA-R by its subunit composition could be said to correlate, in laminar fashion, with the relative exposure to backward connections.

Studies with pharmacological manipulation of NMDA-R in vivo are rare. However, application of an NMDA-R agonist to cat V1 raised the gain of response to stimulus contrast (Fox et al. 1990). The effect was observed in all layers, except layer 4. Application of an NMDA-R antagonist had the reverse effect, reducing the gain such that the contrast response curve (now mediated by non-NMDA-R) became more linear. However, the gain-reduction effect was only observed in layers 2 and 3. To interpret these results, the NMDA-R agonist may have simulated a recurrent enhancement of responses in the layers exposed to backward connections (i.e. all layers save layer 4). The experiments were conducted under anaesthesia, minimising activity in backward pathways, and hence restricting the potential to observe reduced gain when applying the NMDA-R antagonist. The restriction of the antagonist effect to layer 2/3 could indicate that NMDA-R plays a more significant role in nonlinear intrinsic processing in these layers (e.g. in mediating direction selectivity, see Rivadulla et al. 2001). The relative subunit composition of NMDA-R in cat V1 is not known.

Finally, the modulatory properties of backward connections have been demonstrated at the level of the single neuron. The mechanism depends on the generation of ‘NMDA spikes’ within the thinner, more distal ramifications of basal and apical dendrites (Larkum et al. 2009; Schiller et al. 2000), whose capacity to initiate axonal spikes is potentiated through interaction with the backpropagation of action potentials from the axon hillock through to the dendritic tree. The effect was demonstrated for apical dendrites in layer 1, and could simulate a backward connection enhancing the gain of a neuron and allowing coincidence detection to transcend cortical layers (Larkum et al. 1999, 2004, 2009).

Note, also, that in highlighting the modulatory character of backward connections we are not assuming a total lack of the driving capability inferred from the in vitro studies (Covic and Sherman 2011; De Pasquale and Sherman 2011). For instance, the NMDA mechanism for pyramidal neurons described above might, potentially, be self-sustaining once initiated. Imaging studies of top-down influences acting on area V1 imply that backward connections can sustain or even initiate activity, in the absence of a retinal signal (e.g. Muckli et al. 2005; Harrison and Tong 2009). This is important from the point of view of predictive coding because, as noted above, top-down predictions have to drive cells that explain away prediction error. From a computational perspective, the key role of modulatory effects is to model the context-sensitive and nonlinear way in which causes interact to produce sensory consequences. For example, backward projections enhance the contrast between a receptive field’s excitatory centre and inhibitory surround (Hupé et al. 1998).

A summary of the laminar, topographic and physiological characteristics of forward and backward connections in the visual system can be found in Table 1. These characteristics are now be used as tests of directionality for descending projections in the motor system.

## Motor projections

In this section, we summarise the evidence that suggests descending connections in the motor system are of a backward type and are therefore in a position to mediate predictions of proprioceptive input. See Fig. 9 for a schematic of the implicit active inference scheme. As noted above, these predictions rest upon context-sensitive and implicitly nonlinear (modulatory) synaptic mechanisms and are broadcast over divergent descending projections to the motor plant.

### Laminar characteristics

Prior to a detailed examination of motor cortex—BA 4 and BA 6—two well-known features are worth noting. The first is the regression of the ‘granular’ layer 4, that is commonly described as absent in area 4—although Sloper et al. (1979) clearly demonstrated a layer 4 in macaque area 4 as a diffuse middle-layer stratum of large stellate cells—or present as an ‘incipient’ layer in parts of area 6; sometimes referred to as dysgranular cortex (Watanabe-Sawaguchi et al. 1991). The second feature is that the deep layers 5 and 6—the source of massive motor projections to the spinal cord—are around twice the thickness of the superficial layers 1–3 (Zilles et al. 1995). These projections originate in large pyramidal cells (upper motor neurons, including Betz cells) in layer 5. These differences in the architecture of motor cortex clearly suggest an emphasis on the elaboration of backward rather than forward connections—but the relative absence of layer 4 implies that the laminar rules developed for sensory cortex cannot be applied without some modification.

**Fig. 4:**
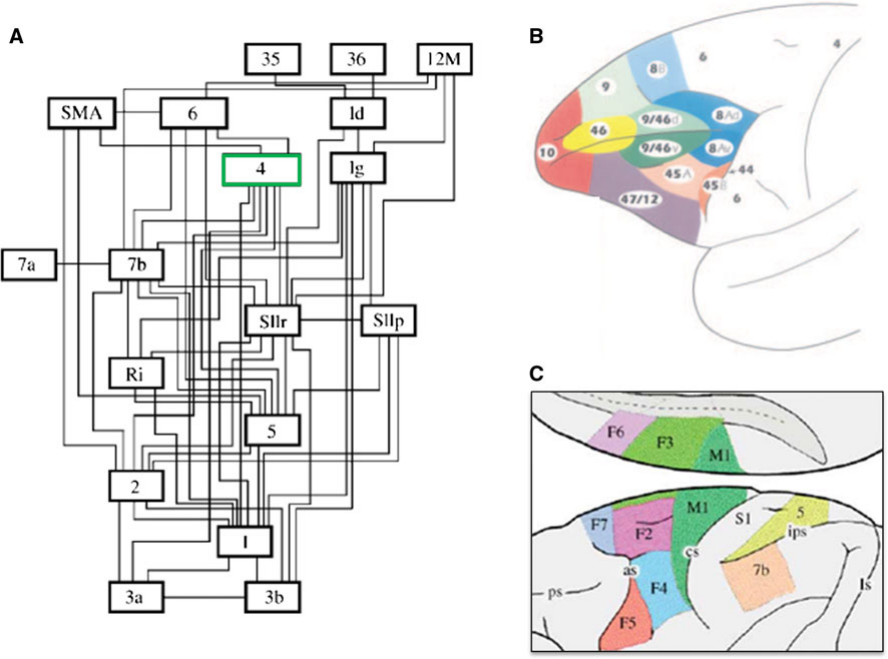
Somatomotor hierarchy and anatomy. **a** The somatomotor hierarchy of Felleman and Van Essen (1991), with several new areas and pathways added by Burton and Sinclair (1996). *Ri*, *Id* and *Ig* are in the insula, *35* and *36* are parahippocampal, and *12M* is orbitomedial. The key point to note here is the high level of M1 (Brodmann’s area 4 in *green*) in the hierarchy. **b** Prefrontal areas in the macaque, taken from Petrides and Pandya (2009). The frontal motor areas have been *left white*, and are illustrated in the figure below. **c** Somatomotor areas in the macaque, adapted from Geyer et al. (2000). Areas *F2*, *F4*, *F5* and *F7* constitute premotor cortex, and *F3* and *F6* the supplementary motor area (*SMA*) together they form area 6. Primary motor cortex (*M1*) is area 4, primary sensory cortex (*S1*) areas 1–3, and areas 5 and 7b are secondary sensory areas. *ps*, *as*, *cs*, *ips* and *ls* are principal, arcuate, central, intraparietal and lunate sulci, respectively

**Fig. 5:**
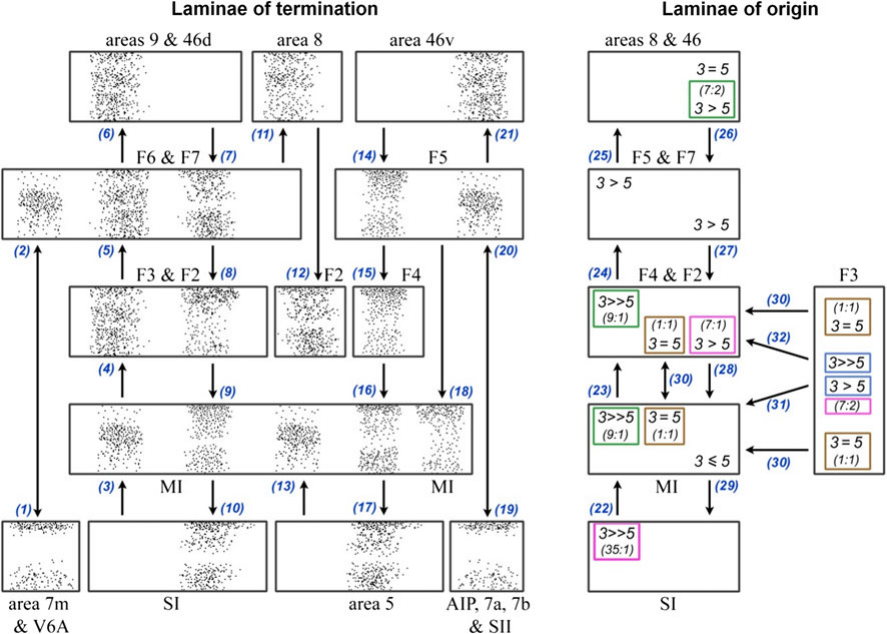
Laminar systematics in the somatomotor hierarchy: this figure is updated from Shipp (2005). The diagrams show patterns of terminations (*left*) and cells of origin (*right*) in selected areas comprising the somatomotor hierarchy (shown anatomically in Fig. 4b, c). Not all connections are shown, only those for which an adequate indication of laminar characteristics is obtainable (the *blue numbers* provide a key to the literature). In order to compile data across studies with variable terminology and placement of injected tracers, or with similar outcomes, some areas are combined into single blocks; the *ampersand* should be interpreted as ‘and/or’. The diagrams are intended to give an indication of forward or backward relationships, but not the precise number of pathways or levels involved. The sensory tiers, for instance, are compressed into a single level: *S1* shown as a single block, comprises four separate areas (3a, 3b, 1 and 2) that precede higher order parietal areas in a sensory hierarchy. *Left panel* schematic illustrations of terminal patterns—forward (2, 3, 13 and 20); intermediate (4, 5, 6, 11 and 21); and backward (1, 7–10, 12 and 14–19). Forward patterns have a concentration in layer 3. Intermediate patterns are described as columnar, with little or no laminar differentiation. Backward patterns are concentrated in layers 1 (and 6) and/or tend to avoid the lower part of layer 3. Feedback from M1 to S1 tends to avoid layer 4. *Right panel* laminar distribution of cells of origin, coded as the relative density of labelled cells in layers 3 and 5. In general, ascending connections are associated with a high 3:5 ratio, and descending connections with a lower 3:5 ratio (that may still exceed unity). Factors influencing cell density can vary considerably across studies and few provide quantitative cell count data. *Coloured boxes* emphasise four studies that provide comparative cell data for connections at two or more separate levels. *Pink* the ascending input to M1 from S1 has a greater 3:5 ratio than the descending input to M1 from premotor cortex (data from Ghosh et al. 1987). *Green* the ascending and descending inputs to premotor cortex show a similar relationship (Barbas and Pandya 1987). *Brown* a study in which the interconnections of M1 with premotor and supplementary motor cortex were not found to be distinct (Dum and Strick 2005). *Blue* the depth profile of connections from F3 (area SMA) to M1 and to premotor cortex were shown to differ, neurons projecting to M1 being less superficial (Johnson and Ferraina 1996). There are no quantitative data where the density of layer 5 cells much exceeds layer 3 cells in motor connections, and only rare qualitative descriptions to this effect, e.g. for the projection from F4 to M1 (Stepniewska et al. 1993); and from M1 to area 1 (Burton and Fabri 1995). *1* Künzle (1978a); *2* Jones et al. (1978), Shipp et al. (1998), Leichnetz (2001); *3* Jones et al. (1978), Künzle (1978b), Pons and Kaas (1986); *4* Künzle (1978b), Leichnetz (1986), Matelli et al. (1986), Stepniewska et al. (1993); *5* Barbas and Pandya (1987); *6* Künzle (1978a), Barbas and Pandya (1987); *7* Watanabe-Sawaguchi et al. (1991); *8* Künzle (1978a), Barbas and Pandya (1987), *9* Barbas and Pandya (1987), Watanabe-Sawaguchi et al. (1991); *10* Jones et al. (1978), Künzle (1978b), Leichnetz (1986), Stepniewska et al. (1993); *11* Künzle (1978a), Barbas and Pandya (1987), Watanabe-Sawaguchi et al. (1991); *12* Arikuni et al. (1988); *13* Jones et al. (1978), Pons and Kaas (1986); *14* Preuss and Goldman-Rakic (1989), Watanabe-Sawaguchi et al. (1991); *15* Künzle (1978a), Barbas and Pandya (1987), Deacon (1992); *16* Künzle (1978b), Watanabe-Sawaguchi et al. (1991); *17* Künzle (1978b), Leichnetz (1986); *18* Barbas and Pandya (1987); *19* Künzle (1978a), Matelli et al. (1986), Barbas and Pandya (1987), Deacon (1992), Gerbella et al. (2011); *20* Rozzi et al. (2006), Borra et al. (2008); *21* Barbas and Pandya (1987), Deacon (1992), Gerbella et al. (2011); *22* Jones et al. (1978), Leichnetz (1986), Ghosh et al. (1987), Huerta and Pons (1990), Darian-Smith et al. (1993), Stepniewska et al. (1993); *23* Matelli et al. (1986), Barbas and Pandya (1987), Kurata (1991), Watanabe-Sawaguchi et al. (1991); *24* Barbas and Pandya (1987), Deacon (1992); *25* Arikuni et al. (1988), Watanabe-Sawaguchi et al. (1991), Lu et al. (1994); *26* Barbas and Pandya (1987), Watanabe-Sawaguchi et al. (1991), Deacon (1992), Gerbella et al. (2011); *27* Kurata (1991); *28* Muakkassa and Strick (1979), Godschalk et al. (1984), Leichnetz (1986), Ghosh et al. (1987), Stepniewska et al. (1993), Lu et al. (1994); *29* Pons and Kaas (1986), Darian-Smith et al. (1993), Burton and Fabri (1995); *30* Dum and Strick (2005); *31* Muakkassa and Strick (1979), Godschalk et al. (1984), Leichnetz (1986), Ghosh et al. (1987), Stepniewska et al. (1993), Lu et al. (1994), Johnson and Ferraina (1996); *32* Matelli et al. (1986), Kurata (1991), Johnson and Ferraina (1996)

Shipp (2005) performed a literature analysis of the laminar characteristics of projections in the motor system, motivated by the “paradoxical” placement of area 4 (primary motor cortex) *below* area 6 (premotor cortex) and the supplementary motor area in the Felleman and Van Essen (1991) hierarchy (Fig. 4a). Note that this placement is only paradoxical from the point of view of conventional motor control models; it is exactly what is predicted by active inference. The schematic summary of this meta-analysis is reproduced here, with some additions and updates (Fig. 5).

**Fig. 6:**
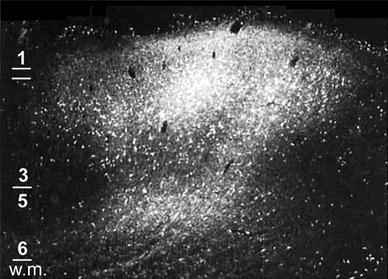
Backward termination pattern of a premotor to M1 projection: Adapted from Watanabe-Sawaguchi et al. (1991), this is a darkfield photomicrograph showing labelled cells and terminals in area 4 after injection of WGA-HRP into the inferior premotor area (PMv, or F5) of a baboon. The termination pattern is characteristic of a backward connection as it is bilaminar (with a particularly dense supragranular projection) and minimally dense in lower layer 3. *W.m.* signifies white matter

The scheme includes connections originating in primary and higher order sensory cortex, primary motor cortex, subdivisions of premotor and supplementary motor areas and areas of prefrontal cortex just rostral to motor cortex, arranged in a hierarchy according to the characteristics of forward and backward connections in Table 1. Following Felleman and Van Essen (1991), forward connections to agranular cortex are identified with terminal concentrations in layer 3, as ascending terminations in sensory cortex typically terminate in both this layer and layer 4 (see also Rozzi et al. 2006; Borra et al. 2008). Conversely, backward-type terminations in agranular cortex can be characterised by avoiding layer 3, and/or being concentrated in layer 1 (see Fig. 6).

To what extent do corticocortical motor projections conform to the forward/backward tests? We list the major findings, followed by a more forensic analysis.

- The terminations of projections ascending the somatomotor hierarchy are intermediate in character (terminate in all layers) apart from those originating in the sensory areas of parietal cortex, which have the characteristics of forward projections.
- The terminations of projections descending the somatomotor hierarchy have an overall backward character. The pattern is notably more distinct for terminations within postcentral granular areas, but the available evidence leans toward a backward pattern in the precentral agranular areas as well.
- The origins of projections ascending or descending the somatomotor hierarchy are qualitatively similar to each other; the projecting neurons are typically described as bilaminar and equally dense in layers 3 and 5, or as predominating in layer 3.

Regarding (c), the proposition that both ascending and descending connection originate primarily from layer 3 breaches the rules of forward and backward connectivity developed for sensory cortex (Table 1). However, there is considerable variability in the reported laminar density of neurons that are labelled with retrograde tracers (attributable to factors such as the type of tracer used, its laminar spread at the site of deposition, survival time, and the means of assessment). To circumvent such problems, Fig. 5 emphasises quantitative data (the layer 3:5 ratio) obtained for two or more projections in the same study, thus enabling a more robust comparison of ascending and descending connections assessed with identical methodology. This ‘ratio of ratios’ approach suggests that the origin of ascending projections within the somatomotor hierarchy may be characterised by a higher superficial: deep ratio than the origin of descending connections, even if both ratios are above one. This is true for (1) projections to M1 from S1 versus premotor cortex (PMd), and (2) projections to PMd from M1 versus rostral frontal cortex. The ratio of ratios device may depart from the original test criteria but as Felleman and Van Essen (1991) point out: “the key issue is whether a consistent hierarchical scheme can be identified using a modified set of criteria”.

Notably, both the above examples involve a comparison stretching across three hierarchical levels; when direct reciprocal connections are examined between areas on notionally adjacent levels; i.e. between M1 and PMd, PMv or SMA, the patterns of retrograde labelling are reportedly broadly similar (Dum and Strick 2005). This more recent study holds that motor, premotor and supplementary motor interconnections all show an ‘equal’ pattern of superficial: deep cell labelling (i.e. % superficial within 33–67 %), associated with a ‘lateral’ connection in hierarchical terms. The discrepancy with the earlier cell-count data may reflect methodological differences, but can also be given a more systematic interpretation: that, similar to sensory cortex, the laminar patterns associated with the motor hierarchy obey the ‘distance rule’ (Barone et al. 2000), and are more marked when assessing connections over a larger number of levels.

If the laminar origins of directly reciprocal projections are similar, a different style of analysis might be needed to reveal differences. An example is a study by Johnson and Ferraina (1996), who noted that cells in SMA projecting to PMd were more concentrated in the superficial layers than cells projecting from SMA to M1: they used a statistical comparison of the mean and shape of the two depth distributions to confirm that the difference was significant. In summary, the available evidence suggests ascending connections in the motor system have a forward character and descending connections are backward in nature. There is no evidence for the reverse. The bilaminar origins of motor connections indicate that motor, premotor and supplementary motor cortices are close together in the somatomotor hierarchy.

In sensory cortex, it is generally accepted that bilaminar origins can be consistent with forward, lateral, or backward projections, and that patterns of termination are typically more indicative of hierarchical order (Felleman and Van Essen 1991). The motor system may be similar, but as relatively few adequate descriptions of laminar terminal patterns are available, the indications derive from an uncomfortably small number of reports. Ascending projections are typically described as being columnar—a multilayer distribution that would be consistent with a lateral connection. Perhaps the best documented example is the projection from M1 to SMA, illustrated by photomicrographs in three separate studies (Künzle 1978b; Leichnetz; Stepniewska et al. 1993). Künzle (1978b) noted: “the anterograde labelling within the columns appeared somewhat heavier in supragranular layers 1–3 than in infragranular layers 5–6”, whilst Stepniewska et al. (1993) put it thus: “anterogradely labelled axons and terminals are concentrated mainly in layers 1 and 3–6, leaving layer 2 almost free of label”. The material obtained by each study is clearly comparable, and does not readily demonstrate forward characteristics. The projections to agranular cortex that do display a forward pattern; i.e. terminating mainly in the mid-layers, are those arising in sensory cortex, e.g. from areas 2 and 5 to M1, or from several visuosensory parietal areas to premotor cortex (see Fig. 5 for references).

Backward laminar patterns for motor and premotor projections are most evident in parietal cortex, typified by the following description: “an unlabeled line highlighted lamina 4 amid substantial anterograde labelling in the supra- and infragranular layers above and below it” (Leichnetz 1986). For motor cortex itself, there is just one equivalent description, pertaining to a back projection from area F4 to M1, where “the labelled terminals were distributed throughout all layers, with the exception of the lower half of layer 3″ (Watanabe-Sawaguchi et al. 1991); this connection is reproduced here in Fig. 6. The backward pattern is alternatively diagnosed by a superficial concentration of terminals, especially within layer 1—e.g. “in certain regions, such as area 4… label was found predominantly in supragranular layers and especially in layer 1” (Barbas and Pandya 1987). Künzle (1978a), also describing premotor cortex projections, makes a similar comment: “the cortical projections are found to terminate consistently and often with highest intensity within cortical layer 1″. This description was a global one, including occipital and parietal cortex where the layer 1 concentration may have been most prominent. However, his sketches of terminations within motor cortex show several connections that appear to satisfy this description, again as listed in Fig. 5. It is possible that motor terminal patterns also observe the ‘distance rule’ and are more liable to display hierarchical character when they traverse more than one level; this could apply to the cases illustrated by Barbas and Pandya (1987), for instance.

The literature survey lacks detailed studies of reciprocal terminal connections, examined area by area with identical methods. If, as we infer, a lateral (multilaminar) pattern of terminals in the ascending direction is reciprocated by a backward pattern in the descending direction, this infringes on the standard hierarchical dogma (which would hold that a pair of reciprocal connections should both be ‘lateral’, or that one should be forward and the other backward (Felleman and Van Essen 1991). The anomaly might be rectified by an appropriate, purposeful study of reciprocal terminal connections in motor cortex. Alternatively, the standard dogma might simply fail to address the full diversity of cortical connectivity; other factors, such as differential architecture, may also be determinants of laminar patterns (Barbas 1986; Hilgetag and Grant 2010). Ultimately, the study of laminar patterns is a proxy for a more sophisticated, physiological determination of the functional composition of a projection; e.g. as characterised by driving or modulatory synaptic contacts (Covic and Sherman 2011) on particular subclasses of excitatory or inhibitory neurons (Medalla and Barbas 2009).

In the absence of such evidence, the interim conclusion is that laminar connectivity patterns reveal a relatively clear-cut hierarchical divide within the somatomotor system between (higher) precentral agranular motor and (lower) postcentral granular sensory areas, and that hierarchical divisions within the agranular areas are more subtle. Even so, the indications from both origins and terminations place rostral premotor (or even prefrontal) cortices at the apex of the somatomotor hierarchy, favouring the active inference model over a serial motor command model.

### Topographic characteristics

In the sensory system, backward connections widely bifurcate and have very distributed terminations, and are both more divergent and more convergent than their forward counterparts (see Fig. 3). Do descending connections in the motor system share these properties?

**Fig. 7:**
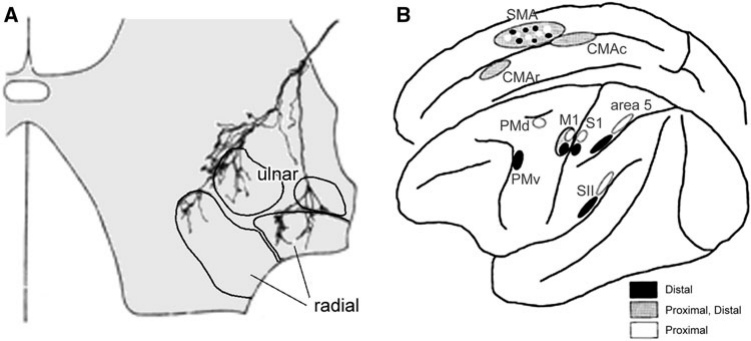
Topographic characteristics of projections in the motor system: **a** adapted from Shinoda et al. (1981), this is a transverse section through the spinal cord at level C7, showing a corticospinal axon that projects to at least four different motor nuclei: those of the ulnar (the upper nucleus), radial (the lower two nuclei) and medial nerves (not shown). **b** Adapted from Tokuno and Tanji (1993), this depicts cortical areas containing neurons projecting to proximal (*white*) and distal (*black*) movement areas of M1. Lower hierarchical areas have segregated projections, whereas higher projections are intermixed (*grey*) with the exception of premotor cortex, whose inputs were subsequently also shown to be intermixed (Dancause et al. 2006). *CMAc* caudal cingulate motor area, *CMAr* rostral cingulate motor area, *MI* primary motor cortex, *PMd* dorsal premotor cortex, *PMv* ventral premotor cortex, *SI* primary somatosensory cortex, *SII* secondary somatosensory cortex, *SMA* supplementary motor area

First consider the connections of motor neurons in the periphery. A single motor neuron innervates hundreds of muscle fibres, and these fibres do not form discrete clusters but are distributed uniformly across part of a muscle (a motor unit). There is therefore extensive overlap of the motor units innervated by different motor neurons (Schieber 2007). Further divergence on the microscopic level is shown by corticospinal axons: one studied by Shinoda et al. (1981) innervated motor neurons in the nuclei of the radial, ulnar and median nerves (Fig. 7a), and neurophysiological evidence for this anatomical divergence was found by Lemon and Porter (1976).

The distribution of corticospinal neurons innervating (via spinal motor neurons) a single muscle can be examined by use of the rabies virus tracer; this subset of corticospinal neurons which synapse directly with spinal motor neurons, as opposed to interneurons, are known as ‘CM’ neurons (described at length in the “Discussion”). Comparison of cases examining digit, elbow and shoulder muscles reveals the expected gross proximal–distal topography in M1, but also shows intermingling of corticospinal neurons with different targets (Rathelot and Strick 2009). Thus corticospinal axons from a large territory of M1 also *converge* on a single body part (also see Geyer et al. 2000).

A complementary set of cortical ‘muscle maps’ has been obtained by recording electromyographic (EMG) activity within the forelimb musculature, produced across a grid of cortical stimulation sites (Fig. 8b from Boudrias et al. 2010). EMG activity reflects both direct and indirect corticospinal circuitry, and the resulting muscle maps show no sign of segregated regions representing different muscles or muscle groups. Individual stimulation sites commonly yield EMG activity in both proximal and distal muscles, such that the somatotopic organisation of forelimb M1 was only recognisable in the medial and lateral poles of the mapped area with, respectively, proximal and distal segregated muscle representations. In PMd and PMv there was no discernible topography at all, with proximal and distal muscle maps overlapping completely.

**Fig. 8:**
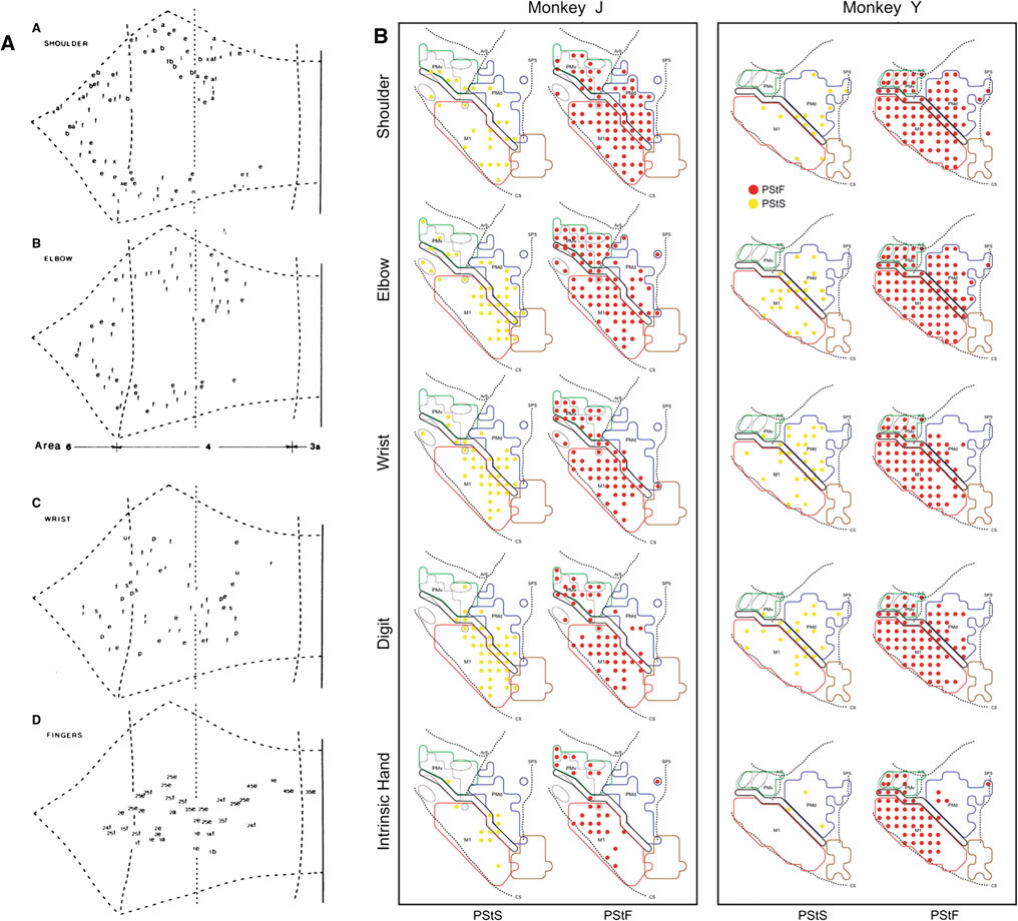
Somatotopic differences in ascending and descending motor projections: this figure illustrates the relative preservation of somatotopy in forward projections and the much greater convergence and divergence in backward projections in the motor system, as is found in sensory projections (schematised in Fig. 3a). **a** This figure is taken from Wong et al. (1978). It illustrates the spatial distribution of neurons in macaque M1 that respond to passive movement of the relevant joint (the tiny letters indicate the direction of movement, not important for our purposes). One can see that the shoulder, elbow, wrist and fingers joints’ representations are overlapping but reasonably somatotopic: non-adjacent joints do not overlap. The 15 % of neurons that responded to movement of multiple joints are not illustrated here. **b** This figure is taken from Boudrias et al. (2010). It illustrates the motor output maps for the premotor cortex and M1 (*top right* and *bottom left* of each drawing, respectively) of two macaques, with each row corresponding to muscles around different joints. The maps were obtained by stimulating in the dotted cortical sites and recording EMGs in peripheral muscles; the red and yellow dots signify post-stimulus facilitation and suppression, respectively. Whilst some resemblance can be seen to the sensory maps in **a**, it is clear that there is far more convergence and divergence of these descending projections

The divergence of M1s neuronal outputs may be contrasted with older data pertaining to their sensory afferent inputs. Neurons responding to joint movement (but not to cutaneous stimulation) are thought to represent sensory input from muscle spindles, and many neurons in M1 are selective for a single joint. For example, Lemon and Porter (1976) found that 110/152 (72 %) of M1 neurons responsive to passive forelimb manipulation in alert macaques were only activated by manipulation of one particular joint (e.g. a single finger joint). The ascending sensory representation of individual muscles in M1 is thus distinctly more focal in nature than the descending (divergent) motor output, as assessed at the level of M1 neurons. At the macroscopic level, Wong et al. (1978) mapped the cortical distribution of these sensory inputs to M1 (Fig. 8a), and found that whilst sensory inputs seem less organised here than in sensory areas (e.g. different sensory modalities innervating the same M1 microcolumns, unlike in S1), there is “virtually no overlap of the… sensory fields related to nonadjacent joints”: in contrast to the M1 motor fields mapped by Boudrias et al. (2010).

Now let us examine corticocortical connections within the sensorimotor system. Studies using dual retrograde tracers have examined the sources of input to the parts of M1 innervating the distal and proximal parts of the monkey forelimb (Tokuno and Tanji 1993) and hindlimb (Hatanaka et al. 2001). They noted that clear, separate subregions in SI, SII and area 5 project to the distal and proximal representations of either limb in M1, whereas the projections from motor areas [cingulate, supplementary and dorsal area 6 (PMd)] were intermixed: several regions in these areas sent axons to both distal and proximal forelimb parts of M1 (Fig. 7b) and a similar pattern was observed for the hindlimb. Essentially, this demonstrates that divergence in the descending (motor) input to M1 exceeds divergence in the ascending (sensory) input to M1.

In a later study, Dancause et al. (2006) used a bidirectional tracer placed in PMv to prepare high-resolution somatotopic maps of the reciprocal corticocortical connections between PMv and M1. Two results are of interest: (a) that the distal forelimb part of PMv connects with both distal and proximal forelimb parts of M1, demonstrating descending divergence similar to the other motor areas noted above; (b) that the termination of the descending projection to M1, while patchy, was broader than the territory occupied by source neurons for the ascending projection, thus replicating the kind of pattern noted previously in visual cortex. Our final test of forward and backward connection types is that if they are of unequal size, backward projections should outnumber forward. In fact, descending projections outnumber ascending ones between area 6 and area 4 (Matelli et al. 1986), areas F6 and F3 (Luppino et al. 1993), and between CMAr and SMA/PMdr (Hatanaka et al. 2003).

### Physiological and pharmacological characteristics

Zilles et al. (1995) demonstrated that human motor cortex has the same distribution of NMDA and non-NMDA receptors as is found elsewhere in the brain: the former are concentrated in supragranular layers, whereas the latter have a uniform (AMPA-R) or infragranular (KA-R) distribution. When a granular layer is present, e.g. in rat prefrontal cortex, NMDA receptors tend to avoid it (Rudolf et al. 1996). Hence, one might expect that as in sensory cortex, descending corticocortical motor connections (terminating in supragranular layers) have access to modulatory synaptic mechanisms.

Ghosh et al. (1987) counted the relative numbers of neurons projecting to monkey forelimb M1. In the 3 animals they examined, 11–31 % of neurons projecting to M1 came from premotor cortex (lateral area 6), whereas 1–17 % of neurons originated in area 5 (higher sensory cortex). Ghosh and Porter (1988) then stimulated these two cortical areas using surface electrodes, and recorded EPSPs and IPSPs in M1. They found that despite the bias in numbers towards descending projections, stimulation of area 5 neurons elicited responses in 90 % of recorded M1 neurons, whereas the same stimulation of premotor cortex caused only 30 % of recorded M1 neurons to respond. One can infer from this that ascending projections from sensory cortex are the more driving in character, despite their lesser number. Likewise, it is known that inactivation of M1 has a more significant effect on the activity in PMv and SMA than vice versa (Schmidlin et al. 2008), which one would expect if descending connections to M1 were more modulatory, and ascending connections from M1 more driving in character.

Shima and Tanji (1998) provide valuable evidence about the receptor types mediating descending connections to M1 from SMA, in comparison to ascending connections to M1 from S1. They showed that 83 % of the M1 neurons activated by stimulation of S1 were suppressed by a non-NMDA-R antagonist, whereas only 10 % were affected by an NMDA-R antagonist. Conversely, of neurons in M1 activated by stimulation of SMA, roughly equal proportions (55 %) were affected by NMDA-R and non-NMDA-R antagonists. This indicates that the influence of SMA over M1 depends to a much greater extent upon NMDA-R transmission, that can be nonlinear and modulatory, whilst the ascending connections from S1 to M1 rely more heavily on AMPA or kainate receptors, with linear properties more characteristic of driving connections.

Shima and Tanji (1998) speculated that SMA—via NMDA-Rs—might modulate the gain of driving S1 inputs to M1. Evidence for higher motor areas modulating the gain of M1 neurons has actually been provided by Shimazu et al. (2004), who recorded corticospinal outputs following stimulation of the ventral premotor area (F5) and/or M1. M1 stimulation alone evoked several corticospinal volleys, whereas F5 stimulation alone evoked minimal output. If F5 stimulation directly preceded that of M1, however, the later corticospinal volleys were powerfully facilitated, as were the resulting EPSEs in 92 % of intrinsic hand motor neurons. A similar outcome was observed when the experiment was repeated in an alert monkey performing a motor task, allowing the additional observation that the effect of F5 stimulation varied with the type of grasp being performed (Prabhu et al. 2009).

Finally, in relation to descending corticospinal projections, note that cortical modulatory connections have smaller EPSPs which show facilitation with stimulus repetition and are more non-linear: the direct synapses of corticospinal neurons with motor neurons also have these properties. Their unitary (single fibre) EPSPs are of the order of 25–120 μV (Asanuma et al. 1979; Porter 1985): much less than corticocortical unitary EPSPs which are more often >1 mV (Avermann et al. 2012; Andersen et al. 1990; Sáez and Friedlander 2009; Zilberter et al. 2009). Furthermore, on repeated corticospinal stimulation, the motor neuron unitary EPSPs show facilitation (Jankowska et al. 1975; Shapovalov 1975).

It is also established that the targets of corticospinal projections express NMDA receptors: for instance, spinal interneurons and Renshaw cells (McCulloch et al. 1974; Lamotte d’Incamps and Ascher 2008) and also motor neurons (Tölle et al. 1993), which have been shown to contain the more non-linear NR2B subunit (Mutel et al. 1998; Palecek et al. 1999). This provides a synaptic mechanism for the contextual (nonlinear gain control) nature of descending predictions from corticospinal motor neurons. Note that neither conventional motor control models nor optimal control schemes would predict that corticospinal projections should have modulatory properties (as a motor command must be driving, not modulatory). Active inference, by contrast, allows a mixture of modulatory and driving capabilities in its descending projections, which (as noted in the previous section) can both be compatible with backward connections.

A summary of our analysis can be found in Table 1. It is clear that with some minor adjustment of the criteria for forward and backward connections, ascending and descending connections in the motor hierarchy should be classified as forward and backward types, respectively. This supports our contention that somatomotor system may implement active inference, in which backward connections provide predictions and forward connections convey prediction errors.

## Discussion

We started by motivating the importance of classifying motor efferents as forward or backward by appealing to the competing theoretical predictions of active inference and conventional motor control. The weight of empirical evidence suggests that descending connections in the somatomotor system are of the backward type, as would be required by active inference. In this section, we review the anatomical implications of active inference for the functional anatomy of the motor system, including the unique cytoarchitectonic feature of motor cortex (Brodmann’s area 4 and area 6): its curious regression of a granular layer 4.

### Active inference and sensory reafference

**Fig. 9:**
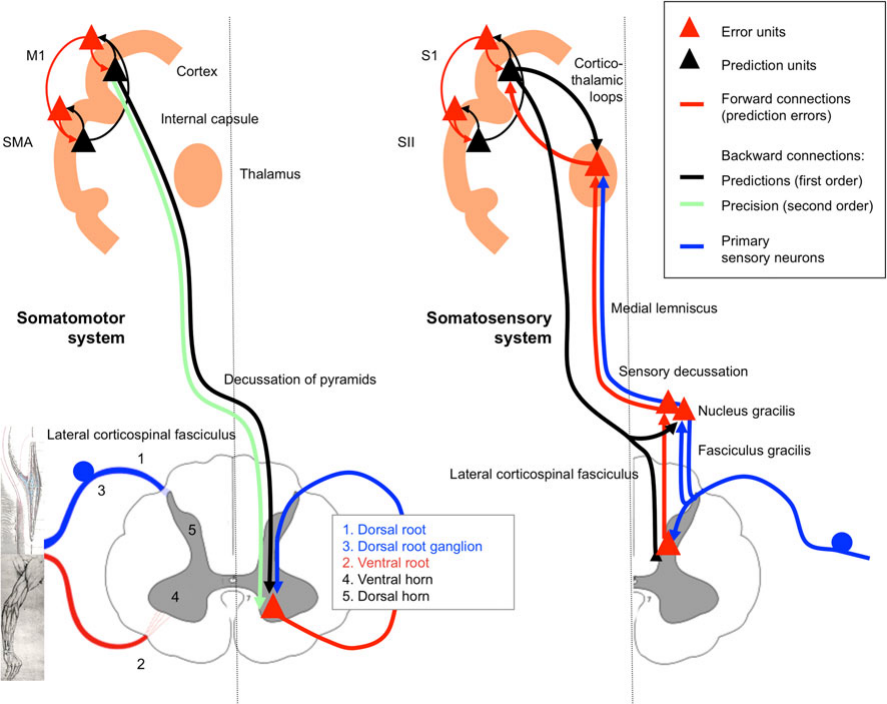
Somatomotor and somatosensory connections in active inference: In this figure, we have focused on monosynaptic reflex arcs and have therefore treated alpha motor neurons as prediction error units. In this scheme, descending (corticospinal) proprioceptive predictions (from upper motor neurons in M1) and (primary sensory) proprioceptive afferents from muscle spindles converge on alpha motor neurones on the ventral horn of the spinal cord. The comparison of these signals generates a prediction error. The gain of this prediction error is in part dependent upon descending predictions of its precision (for further explanation see ‘CM neurons and predictions of precision’ in the “Discussion”). The associated alpha motor neuron discharges elicit (extrafusal) muscle fibre contractions until prediction error is suppressed. Ascending proprioceptive and somatosensory information does not become a prediction error until it encounters descending predictions, whether in the (ventral posterior nucleus of the) thalamus, the dorsal column nuclei, or much earlier in the dorsal horn. In the cortex, error units at a given level receive predictions from that level and the level above, and project to prediction units at that level and the level above (only two levels are shown). In this way, discrepancies between actual and predicted inputs—resulting in prediction errors—can either be resolved at that level or passed further up the hierarchy (Friston et al. 2006). Prediction units project to error units at their level and the level below, attempting to explain away their activity. Crucially, active inference suggests that both proprioceptive (motor) and somatosensory systems use a similar architecture. It is generally thought that prediction units correspond to principal cells in infragranular layers (deep pyramidal cells) that are the origin of backward connections; while prediction error units are principal cells in supragranular layers (superficial pyramidal cells) that elaborate forward projections (Mumford 1992; Friston and Kiebel 2009). Note that we have implicitly duplicated proprioceptive prediction errors at the spinal (somatomotor) and thalamic (somatosensory) levels. This is because the gain of central (somatosensory) principal units encoding prediction error is set by neuromodulation (e.g. synchronous gain or dopamine), while the gain of peripheral (somatomotor) prediction error units is set by NMDA-Rs and gamma motor neuron activity. In predictive coding, this gain encodes the precision (inverse variance) of prediction errors (see Feldman and Friston 2010). Algorithmically, the duplication of prediction errors reflects the fact that somatomotor prediction errors drive action, while somatosensory prediction errors drive (Bayes-optimal) predictions. For reasons of clarity we have omitted connections ascending the cord in the somatomotor system, e.g. spinal projections to M1 and the transcortical reflex pathway from S1 (in particular the proprioceptive area 3a) to M1: these are described in the “Discussion”

From the point of view of active inference, motor cortex occupies a relatively high level in a predictive coding hierarchy (see Fig. 4a), providing predictions of sensory input to several subordinate structures, ranging from spinal circuits to sensory cortex. The graphical representation of this view, shown in Fig. 9, highlights the distinction between somatomotor prediction errors which result in movement, and somatosensory prediction errors that inform percepts. In the somatomotor system (left panel), descending corticospinal projections encode predictions of proprioceptive input; i.e. muscular (muscle spindle), tendon (Golgi tendon organ) and articular states. Together, these signals predict the sensory consequences of a movement *trajectory*; i.e. changes in proprioception or kinaesthesia during the course of the movement and the proprioceptive state at its conclusion. Comparison of these predictive signals with the proprioceptive states encoded by sensory receptors generates proprioceptive prediction errors that—uniquely in the nervous system—*can be resolved by action*, via activation of alpha motor neurons in the spinal reflex arc. The consequences of these actions, both proprioceptive and somatosensory, are then transmitted back to sensorimotor cortex as various forms of sensory reafference.

The descending projections from motor cortex to the somatosensory system (not shown) encode predictions of a broader set of afferent inputs: proprioception plus cutaneous sensations (pressure receptor and light touch receptor states). Because it generates predictions in both proprioceptive and exteroceptive modalities, motor cortex can thus be regarded as a multimodal sensorimotor area rather than a purely motor area (Hatsopoulos and Suminski 2011).

Sensory reafference can become sensory prediction error at various levels in the nervous system (right panel, Fig. 9). The majority of afferents interact at an early stage with corticospinal input to the dorsal horn (Lemon and Griffiths 2005), where prediction errors can be generated by the presynaptic inhibition of primary afferents (Wall and Lidierth 1997). Lemon and Griffiths (2005) suggest, in fact, that this predictive modulation of sensory input is the evolutionary precursor to direct corticospinal control of motor neurons (see below). The remaining primary afferents in the dorsal columns (15 % of the fibres) may then encounter descending predictions at the level of the dorsal column nuclei (via branches of corticospinal axons (Cheema et al. 1985; Bentivoglio and Rustioni 1986), and subsequently at the thalamus. Note that the corticospinal tract is one likely source of the attenuation of spinal sensory reafference during movement (also seen in sensorimotor cortices); uniquely, the sensory reafference to M1 is also inhibited during motor planning (Seki and Fetz 2012).

Proprioceptive reafference to precentral motor cortex (not shown) is conveyed via the spinothalamic tract, which projects to the motor thalamus (the ventral lateral posterior thalamic nucleus, VLp)^3^ and then to primary motor cortex (Stepniewska et al. 2003). Retrograde tracing techniques show that the origin of spinothalamic inputs to VLp are separate clusters of interneurons sited in layers V and VII of the spinal grey matter (Craig 2008). Both groups are thought to integrate primary afferent sensory signals with descending motor signals—layers V and VII processing cutaneous and proprioceptive signals, respectively—which Craig succinctly summarises as an ascending projection “conveying activity that represents the state of the segmental interneuronal pools that are used for motor control”. Another pathway likely to carry ascending proprioceptive prediction errors is the set of dorsal spinocerebellar tract neurons that constitute the thoracolumbar nucleus known as ‘Clarke’s column’. This nucleus, known to receive proprioceptive input from the hind limb, has recently been shown (in the mouse) to interact with signals carried by descending corticospinal axons (Hantman and Jessell 2010); the interaction has both excitatory and inhibitory components (mediated by local interneurons), which could potentially generate a proprioceptive prediction error. Once again, these signals reach motor cortex via VLp (not shown in Fig. 9).

Figure 9 is intended to highlight the distinction between somatomotor prediction errors that result in movement, and somatosensory prediction errors that inform percepts. Of course, the distinction between the somatomotor and somatosensory systems themselves is not so easily made: as we mention above, motor cortex might best be regarded as a multimodal sensorimotor area. One could view some sensory cortices in the same light: for example, the border (proprioceptive) area 3a likely receives descending proprioceptive predictions from M1 (Witham et al. 2010)—rather than efference copy—and ascending proprioceptive information from the motor nuclei of the thalamus (Huffman and Krubitzer 2001). At the same time it is embedded within the somatosensory system, receiving somatosensory thalamic input. Somatomotor (proprioceptive) prediction errors in this area could either be resolved by movement via projections to gamma motor neurons (see next section), or they could inform proprioception via projections to secondary sensory cortices. Likewise, it is notable that S1 cortex drives whisker retraction in the mouse (Matyas et al. 2010).

### Corticomotor (CM) neurons and predictions of precision (gain)

As noted above, the beauty of the spinal reflex arc is that proprioceptive prediction errors can be resolved simply, quickly and automatically by agonist and antagonist muscles. But there is an additional longer-latency, transcortical component to many reflexes (particularly hand and finger reflexes) that is known to exhibit a higher degree of intermuscular coordination, thereby being ‘more intelligent than reflexive’ according to some authors (Matthews 1991; Kurtzer et al. 2008; Shemmell et al. 2010; Pruszynski et al. 2011). Neurons in motor thalamus (VLp) and motor cortex are known to be capable of short latency sensory responses to limb movement (Herter et al. 2009; Hummelsheim et al. 1988; Lemon and Porter 1976; Vitek et al. 1994) although the particular anatomical pathway providing short latency input has been difficult to establish, as there is no anatomical confirmation of lemniscal input to VLp.^4^ Nonetheless, however mediated, this rapid cortical sensory response can be interpreted analogously to the spinal reflex, as a proprioceptive prediction eliciting a motor response, and movement, such as to quash an error signal. How so?

One component of long-latency reflexes is mediated by the specific subpopulation of corticospinal neurons (known as ‘CM’ neurons) that make direct contact with spinal motor neurons, and whose sensory activity (i.e. response to an unexpected torque perturbation of a wrist muscle) formed an appropriate match to the timing of the late (M2) component of the wrist stretch reflex (Cheney and Fetz 1984). The distribution of CM neurons is now known to be quite limited in its extent, occupying the caudal part of M1 and extending into the adjacent component of S1, area 3a (Rathelot and Strick 2006, 2009). CM neurons have their greatest influence upon muscles of the distal forelimb, in both man (de Noordhout et al. 1999; Palmer and Ashby 1992) and rhesus monkeys (McKiernan et al. 1998), and are considered the anatomical substrate for the evolution of manual dexterity in higher primates (Lemon 2008).

CM neurons include both ‘fast’ and ‘slow’ units (as gauged by soma size) and the former, located in M1, likely convey proprioceptive predictions directly to alpha motor neurons. In the case of these direct (AMPA-R mediated) contacts with motor neurons, prediction errors could only be generated by post-synaptic inhibition of those motor neurons by sensory afferent interneurons: see for example the left panel of Fig. 2. These descending fast CM neurons could not themselves carry prediction errors, because they (probably) also synapse with spinal interneurons, implying integration with local sensory input (Kasser and Cheney, 1985): these are descending prediction-type properties, and the same signal cannot be both prediction and error. We propose that the majority of CM neurons, however, mediate a different kind of prediction: not of sensory input itself, but its *precision*.

In predictive coding, there are two kinds of descending predictions (shown in Fig. 9). First order predictions are of sensory input, and therefore drive (or inhibit) prediction error units in the level below, as we described in the first section. Second order predictions are of the precision (inverse variance) of sensory input, and they optimise the post-synaptic gain of the prediction error units below. This is classically a slower process than the first order one, which uses neuromodulators (e.g. NMDA-R’s, acetylcholine or dopamine) rather than fast-on/fast-off transmission (Feldman and Friston 2010). These processes are exactly analogous to the statistical method of weighting the (first order) mean of an experimental observation according to its (second order) standard error: an experimental ‘prediction error’ of high precision will compel a change in the null ‘prediction’. In the cortex, the top-down process of optimising the precision (gain) of prediction error units is called ‘attention’ (Feldman and Friston 2010). Attention should not only optimally increase the gain of sensory signals during perception, but also of proprioceptive signals during movement (Brown et al. 2011).

Two ways in which the precision (gain) of proprioceptive prediction errors can be enhanced are: (1) by increasing the gain of alpha motor neurons via NMDA-Rs, and (2) by increasing the gain of sensory afferents, via the gamma motor neuron drive to intrafusal muscle fibres (see Fig. 2). It is likely that CM neurons fulfil both of these roles [other descending neuromodulatory (e.g. aminergic) systems that we do not review here will also contribute significantly to the gain of prediction errors].

Why do we say that the majority of CM neurons could mediate predictions of precision (gain)? First, this is a possible role for the 15 % of CM neurons located in area 3a, if they project to gamma motor neurons as Rathelot and Strick (2006) surmise, although this has not yet been demonstrated. Second, this could also be the case for the ‘slow’ CM neurons in M1 (the majority), as predictions of precision are slower than first order predictions, as outlined above. Third, it is notable that CM neurons’ EPSPs are greatest in the very places where the (spinal) stretch reflex gain is weakest and most in need of supplementation—the intrinsic muscles of the hand (McKiernan et al. 1998; Ziemann et al. 2004). Last, we would argue that most of the CM system allows the specification of fine-grained, fractionated patterns of motor gain (as well as its first order predictions), in contrast to the diffuse descending neuromodulation found in other systems (Heckman et al. 2008). This proposal integrates observations of the selectivity and focus of CM projections (Buys et al. 1986; Kuypers 1981) with the gain-like qualities listed above. Finally, note that like first order proprioceptive predictions, second order predictions of gain will also be modulated by context, e.g. limb position (Ginanneschi et al. 2005).

### Sensorimotor cortex: granular versus agranular

The concept of an anatomically discrete ‘motor cortex’, localised to the precentral gyrus in anthropoid apes by Sherrington, was first established by Campbell (1905), using the brain of a chimpanzee that had been one of Sherrington’s subjects (Macmillan 2012). Campbell initially studied cerebral myeloarchitecture, noting the prominent wealth of fibres within motor cortex, and although he later included cytoarchitectural features, it was Brodmann’s description of the cytoarchitecture of precentral cortex that gave rise to the description of motor cortex (areas 4 and 6) as ‘agranular’—i.e. lacking the inner granular layer, or layer 4 in his terminology (Brodmann 1909/1994). Although the regression of layer 4 has since been identified as incomplete (Sloper et al. 1979), the gross architecture of motor cortex is evidently highly differentiated from the adjacent postcentral and frontal cortices. And yet, however impressive this may be as a cartographical feature, its functional significance has remained obscure. Why does motor function apparently eliminate (attenuate) the role played by layer 4? With the foregoing discussion in mind, we are now in position to examine the contrasting structure–function relationships within somatomotor cortex, granular S1 versus agranular M1.

The obvious starting point is that loss of layer 4 betrays the absence of a typical ascending pathway, as seen in sensory cortices (Shipp 2005). All the sub-areas of S1 (3a, 3b, 1 and 2), for instance, receive various forms of somatosensory thalamic input in their granular layer (Padberg et al. 2009). In terms of active inference, sensory reafference constitutes prediction errors that serve to correct high-level representations, so refining top-down predictions and leading to sensory percepts. This is a hierarchical process, involving repeated input to layer 4 of the area in a higher tier, and reciprocal feedback of predictions, as we have previously described. Motor cortex activity, by contrast, specifies an intended or predicted movement (goal); this is a fixed entity, relatively resistant to change, except in its fine details or when expectations are violated. Proprioceptive predictions become *fulfilled* in the course of the movement and thus—in the simplest possible state—there is no prediction error to travel over an ascending motor pathway.

This basic intuition has to be qualified, of course, by the existence of the reafferent sensory pathways to M1 that we have noted above, and the fact that connections between motor areas are indeed reciprocal. The next step is therefore to consider how the operations conducted by these pathways may differ from the standard model set by sensory cortex. Clearly, it is misleading to suggest that there is an absence of prediction error reaching motor cortex, and this is not our intention. Anything about the motor environment that is inherently unpredictable (unexpected impacts, deceptively heavy weights, unstable footing, etc.) will cause error in motor predictions, which requires correction. Transcortical reflexes, discussed previously, provide an obvious example. The point to note is that the motor system strategy is not to pass the sensory prediction error up through a chain of cortical areas (as if to modify the intended goal of the movement), but to react rapidly and reissue modified predictions of the intermediate states leading to the same ultimate end state. Let us reiterate the spinal anatomy. M1 does not receive direct afferents from the alpha motor neurons or interneurons that its corticospinal projections target (as would be analogous to the descending projections in a sensory system); rather the proprioceptive reafference percolates through a complex set of spinothalamic and spino-cerebellothalamic circuits, not yet thoroughly documented, but which would seem to offer a wealth of opportunity for it to modify descending predictions at a subcortical level; in other words, the set of spinal and supraspinal reflex arcs that control muscular tension. This forms a rather marked contrast to the more direct route followed by primary afferents along the lemniscal pathway for sensory reafference to S1. This is the message of Fig. 9: the priority of somatomotor prediction errors is to cause movement; the priority of somatosensory prediction errors is to inform percepts.

In passing, it is also important to note that much of the corticothalamic traffic in the motor system involves loops formed with the basal ganglia, and the cerebellum. The former may operate as an action selection system (Gurney et al. 2001), and the latter as an integral part of the forward generative model (see next section). Neither of these loops is operative within sensory systems, and neither may require the fine-grained input analysis associated with a granular layer 4.

### Active inference versus optimal control

So what does the active inference formulation offer, in relation to classical models? One key contribution is to resolve the hard problem of converting desired (expected) movement trajectories in extrinsic coordinates into motor commands in intrinsic coordinates. This hard problem is an ill-posed inverse problem, conventionally ascribed to an inverse model in M1. Active inference dispenses with this hard problem by noting that a hierarchical generative model can map predictions in extrinsic coordinates to an intrinsic (proprioceptive) frame of reference. This means the inverse problem becomes almost trivial—to elicit firing in a particular stretch receptor one simply contracts the corresponding muscle fibre. In brief, the inverse problem can be relegated to the spinal level, rendering descending afferents from M1 predictions as opposed to commands—and rendering M1 part of a hierarchical generative model, as opposed to an inverse model (see Fig. 1).

This division of labour mirrors the distinction made by Krakauer et al. (1999) between the internal (forward) model necessary for computing movement *kinematics* in vectorial coordinates, and the (inverse) model required for computing movement *dynamics*, which takes account of the biomechanical properties of the arm; e.g. interactional torques produced by movement of multiple limb segments. A key difference between our positions is that we locate the inverse mapping in the spinal cord. The location of an inverse model in M1 appeals to evidence that M1 neurons perform computations that are compatible with the outputs of an optimal controller or inverse model; for example, some M1 neurons have been shown to integrate multi-joint torque information (Pruszynski et al. 2011). However, evidence of this sort does not disambiguate between M1 as an inverse model and M1’s pivotal role in a hierarchical generative model. The key distinction is not about mapping from desired states in an extrinsic (kinematic) frame to an intrinsic (dynamic) frame of reference, but the mapping from desired states (in either frame) to motor commands.

Evidence against an inverse mapping occurring in M1 is provided by Raptis et al. (2010), who elicited different EMG patterns following the application of TMS to M1, while the wrist was maintained in flexion or extension positions. If M1 produced motor commands—as an inverse model should—then identical TMS pulses should not elicit the position-dependent EMG patterns observed by Raptis et al (although identical pulses might not produce identical outputs from M1 if the effects of TMS are being modulated by direct proprioceptive feedback to M1). From the point of view of active inference, TMS could be regarded as activating latent (if transient and ill-formed) goals and subsequent predictions—encoded by populations in M1—eliciting position-dependent myoclonic responses, via reflex arcs (and the monosynaptic activation of motor neurons). The crucial point here is that active inference works by providing proprioceptive predictions (from a forward model) to reflex arcs (the inverse mapping), which automatically generate motor commands.

The idea that neuronal activity in motor cortex encodes predicted motor trajectories in extrinsic (3D vectorial) coordinates—as one would expect from a forward model—is supported by studies which extract kinematic information from monkey or human M1 in real time for the control of computer cursors or robotic devices. One of many examples is that of Velliste et al. (2008), who controlled robotic arm movements with electrodes implanted in macaque M1, using the population vector of neuronal activity to represent proprioceptive predictions, from which a robot-derived motor commands to drive movement of its shoulder, elbow and wrist using inverse kinematics. In active inference, this inverse process occurs in the spinal cord—in optimal control, this inversion is assigned to the cortex. Note that correlations between EMG signals and M1 activity (e.g. Cherian et al. 2011) do not necessarily indicate the presence of an inverse model in M1, because these might be expected if CM (and other) M1 neurons mediate predictions of motor precision (gain), as discussed previously.

The circuitry mediating the forward model is potentially rather broad, utilising the cortico-cerebellar thalamic loop that includes not only motor and premotor cortex, but also substantial parts of postcentral cortex, such as area MIP, recently discovered to receive disynaptic input from cerebellum relating to gaze and reach coordination (Prevosto et al. 2010). The potential role of parieto-cerebellar circuitry in a forward model of motor control has been well versed previously (Blakemore and Sirigu 2003; Mulliken et al. 2008). Interestingly, Mulliken et al. (2008) comment that “the encoding of space and time that we observe in posterior parietal cortex may best reflect a visuomotor representation of the [movement] *trajectory*” [emphasis added]: this point supports the active inference view that the generative model must generate movement trajectories, not just end-points.

The initial impetus for the development of forward models in motor control was the realisation that real-time feedback issuing from S1 to M1 would be too slow to influence the control of rapid movements. It also follows that this sensory input to M1 could be of greater importance in motor planning than in online motor control. One way of characterising the interplay between S1 and M1 is that the former models the current body state and the latter the future (intended) body state; if so, the backward connections from motor to sensory cortex could aptly be described as predictions. This could equally include feedback from premotor cortices to superior parietal visual areas, predicting the future location of moving limbs in visual space. A similar argument might account for the fact that as much as 25 % of the corticospinal tract originates from postcentral, sensory areas of cortex (Galea and Darian-Smith 1994). Much of this will represent descending predictions of cutaneous sensation, and may act to cancel or attenuate sensations caused by the body’s own movements, in order to distinguish sensations resulting from external agencies (Blakemore et al. 2000; Cullen 2004).

The above argument is based upon the assumption that pyramidal cells in motor cortex sending predictions to spinal motor neurons (which do not reciprocate a prediction error) are distinct from those sending predictions to somatosensory cortex (which do). This is a sensible assumption in that corticospinal conduction delays require pyramidal cells driving alpha motor neurons to encode the causes of sensory consequences in the near future. Conversely, the principal cells predicting somatosensory consequences in somatosensory cortex have to encode their causes in the recent past. As an aside, these considerations implicitly finesse the problem of conduction delays in motor control by incorporating them into the generative or forward model. This is an established technique in the Bayesian analysis of time series data (Kiebel et al. 2007). One prediction of this separate prospective and retrospective encoding of movements is that the prospective predictions, originating in motor cortex layer 5 pyramidal cells, should not project to sensorimotor cortex or ventrolateral thalamus. In other words, these cells should send direct and monosynaptic connections to the spinal cord and brain stem. This is because the prospective predictions are not suitable for creating predictions of (delayed) sensory input at the thalamic or cortical level. We could find no empirical evidence that refutes this prediction.

### Active inference and complex movements

It may be thought that active inference implies a stimulus-driven account of action. However, most behaviour comprises spontaneous, itinerant movements—like walking and talking. Stimulus-driven behaviours provide intuitive examples of active inference at work, but endogenous and complicated sequences of motor behaviour emerge naturally from priors in hierarchical generative models of movement trajectories. One example—of generating itinerant movement—is that of an agent which learns (and then reproduces) the doodling-type repetitive movements of a Lorentz attractor (Figs 14 and 15 in Friston et al. 2010).

Gestures (especially iconic gestures) are a good example of movements that can be understood under active inference. A related example here is handwriting (handwriting is a difficult behaviour to explain using minimisation of cost functions in optimal control, Friston 2011). Handwriting has been simulated using active inference (Friston et al. 2011), using a simple central pattern generator to produce prior beliefs that an agent’s finger will be drawn to an invisible moving target. An interesting aspect of this simulation was the demonstration that the same central pattern generator was used to infer movement trajectories during *action observation*. In other words, “exactly the same neuronal representation can serve as a prescription for self-generated action, while, in another context, it encodes a perceptual representation of the intentions of another”—as ‘mirror’ neurons do (Friston et al. 2011).

## Conclusion

In conclusion, we have argued that the cortex is best regarded as embodying a hierarchical generative model, whose descending (efferent) projections predict and explain sensory inputs, thus minimising ascending (afferent) prediction errors. This view holds that connections mediating predictions should be more modulatory than those conveying prediction errors, and they should have a similar laminar organisation, irrespective of the sensory modality being predicted. These properties accord well with those of descending projections (from associational to primary cortex) in both sensory and motor systems. This suggests that descending signals in the motor system are not motor commands but proprioceptive predictions—which are realised at the spinal level by classical reflex arcs.

## Appendix 1: Clinical insights from active inference

This appendix highlights some clinical phenomena that shed light on the functional anatomy of motor control. We do not review motor pathology comprehensively but concentrate on areas which could inform—or be informed by—active inference.

### Flaccid and spastic paralyses

Two kinds of paralysis illustrate pathologies of the two types of descending projections in predictive coding networks: those mediating predictions, and those mediating precision (inverse variance of prediction error) or synaptic gain.

A severe neck injury can completely obliterate descending connections from the CNS. The acute consequence is ‘spinal shock’—a flaccid paralysis. In this instance, both proprioceptive predictions (in the corticospinal tract) and predictions of precision (e.g. innervation of gamma motor neurons) are completely lost (Brown 1994). Computationally, the absence of precise proprioceptive prediction errors leads to a loss of lower motor neuron drive and flaccid paralysis.

Conversely, lesions in the cerebral cortex or internal capsule can result in a spastic paresis, in which some muscle power is preserved, but—at rest—patients are hypertonic and hyper-reflexic. In this case, some proprioceptive predictions may reach the ventral horn, but the gain of the stretch reflex is increased due to a loss of supraspinal inhibition. Computationally, proprioceptive prediction errors retain a high precision but are not properly informed by descending predictions—leading to spastic paralysis. It should be noted that an increased stretch reflex gain (mediated by gamma motor neurons) is not the only cause of spasticity; other causes include mechanical changes in the muscle itself (Dietz and Sinkjaer 2007) and increases in the gain of motor neurons (Mottram et al. 2009) following spinal cord injury.

### Proprioceptive deafferentation

One important question for active inference is: if movement depends on spinal reflex arcs, then why can neuropathic patients—who lack Ia afferent feedback from muscle spindles—still move? Surely, in the absence of anything to predict there can be no prediction error and no movement. In fact, the absence of primary afferents does not mean there is no prediction error—top-down predictions can still elicit alpha motor neuron activity. Under active inference, a forward model in the brain converts visuospatial predictions in extrinsic coordinates (low dimensional extrapersonal space) to proprioceptive predictions in intrinsic coordinates (high dimensional proprioceptive space). These predictions then leave the brain and are converted to motor commands by a simple inverse mapping in the spinal cord (see “Discussion”). This spinal inverse mapping is effectively driven by proprioceptive prediction errors and corresponds to the classical reflex arc.

A loss of proprioceptive feedback, therefore, will severely impact upon the spinal inverse mapping, while the cortical forward model can compensate using visual feedback (Bernier et al. 2006). Descending proprioceptive predictions should still be able to activate motor neurons, but they can no longer be compared with precise proprioceptive information and cannot be modified by proprioceptive feedback. These predictions are borne out by empirical studies of deafferented patients:

- Sainburg et al. (1993) showed that two deafferented patients—miming bread slicing with their eyes closed—exhibited severely temporally decoupled movement reversals at the shoulder and elbow joints. Opening their eyes improved the overall form of the movement but inter-joint coordination problems remained.
- Sainburg et al. (1995) demonstrated that the failure to coordinate the timings of movement reversals was due to a failure to compensate for interaction torques transferred to one joint (e.g. the elbow) by changes at another joint (e.g. the shoulder). They concluded that the deafferented patients lacked a model of limb dynamics; i.e. a failure of an inverse mapping.
- Gentilucci et al. (1994) found that a deafferented patient’s first phase of a reaching and grasping movement was identical to that of control subjects, but the final phase required frequent movement adjustment. Furthermore, the patient was unable to compensate for perturbations applied to the fingers, even with visual feedback. This indicates that the patient’s movement planning—i.e. their forward model—is intact, but that they cannot adjust this plan according to limb kinematics, as their inverse mapping is impaired.

### Parkinsonian symptoms

We have previously proposed that neuromodulators—such as dopamine—encode the precision of prediction errors by altering their synaptic gain (Feldman and Friston 2010), and hence their ability to effect change within the network. Depleting dopamine at different levels would alter the balance of precision at higher (sensorimotor) relative to lower (primary sensory) levels in the cortical hierarchy. We have simulated the effects of this loss of precision or gain in previous publications. These vary according to the site (hierarchical level) of changes in precision, and include:

- The emergence of quasi-periodic attractors, manifested as tremor or other repetitive stereotyped movements (Friston et al. 2010). This model of Parkinsonian tremor complements that of Helmich et al. (2012), who propose that Parkinsonian tremor arises from an interaction between striatal dopaminergic depletion and a cerebello-thalamo-cortical circuit (the cerebellum has long been thought to have a role in the temporal coordination of motor signals).
- A loss of precision of proprioceptive predictions resulting in smaller movements (hypokinesia), slower movements (bradykinesia) or loss of movement—akinesia (Friston et al. 2010; Friston et al. 2012).
- A loss of precision of cue salience can preclude set-switching, particularly in the context of a hitherto predictable sequence, resulting in perseveration (Friston et al. 2012)—also shown in Parkinson’s disease (Brown and Marsden 1988).

The phenomenon of hypertonia (rigidity) in Parkinson’s disease is best characterised, conversely, as *increased* high-level gain (as opposed to the precision associated with stretch reflex gain). This could be mediated by a loss of dopaminergic inhibition of striatal cholinergic interneurons—demonstrated in a mouse model of dystonia by Pisani et al. (2006)—but there are likely to be other mechanisms, both spinal and supraspinal (Rodriguez-Oroz et al. 2009).

## Footnotes

1 Another way to put this is that command signals live in a space of motor effectors, whose dimensionality is equal to the number of (extrafusal) neuromuscular junctions—this is the output of the motor neurons. Predictions live in the space of sensory receptors, whose dimensionality is equal to the number of primary afferents—this is input to the motor neurons.

2 As noted by our reviewers, predictions of muscle torque (reported by Ib afferents) might be construed as motor commands, not proprioceptive consequences. The key point here is that the Ib inhibitory interneurons that receive descending predictions do not just receive torque information from Ib afferents, but also inputs from muscle spindles via Ia afferents, articular afferents and low-threshold afferent fibres from cutaneous receptors. It is therefore most accurate to describe the descending prediction of Ib activity as not simply a ‘prediction of torque’, but a ‘prediction of torque in a particular context’. It is this contextual aspect of the prediction that differentiates it from a motor command, which would not be context-dependent. Furthermore, under active inference, actions minimise sensory prediction error not just on position, but also on velocity, acceleration, jerk, smoothness, etc. (Friston et al. 2010). This means proprioceptive predictions will necessarily have a torque component, but they cannot generate this torque: this is the job of the spinal reflex arc.

3 Thalamic terminology follows the scheme of Macchi and Jones (1997).

4 An earlier body of work employing peripheral nerve stimulation provided substantial indirect evidence that sensory input could be conveyed to M1 via dorsal column (lemniscal) input to thalamic VLp nucleus; especially a subnucleus known as VPLo (Asanuma et al. 1980; Horne and Tracey 1979; Lemon and van der Burg 1979). But, conversely, several anatomical studies specifically failed to offer any evidence for such lemniscal input to VPLo (Asanuma et al. 1980, 1983; Hirai and Jones 1988; Kalil 1981; Tracey et al. 1980). The conflict in these observations has yet to be satisfactorily resolved.
